# Core genome sequencing and genotyping of *Leptospira interrogans* in clinical samples by target capture sequencing

**DOI:** 10.1186/s12879-023-08126-x

**Published:** 2023-03-14

**Authors:** Linda Grillova, Thomas Cokelaer, Jean-François Mariet, Juliana Pipoli da Fonseca, Mathieu Picardeau

**Affiliations:** 1 Biology of Spirochetes Unit, French National Reference Centre for Leptospirosis, CNRS UMR 6047, Institut Pasteur, Université Paris Cité, 75015 Paris, France; 2 Institut Pasteur, Université Paris Cité, Plate-Forme Technologique Biomics, 75015 Paris, France; 3 Département Biologie Computationnelle, Institut Pasteur, Université Paris Cité, Hub de Bioinformatique et Biostatistique, 75015 Paris, France; 4 Present Address: Parasites and Microbes Programme, Wellcome Sanger Institute, Hinxton, UK

**Keywords:** Leptospirosis, *Leptospira*, Genotyping, Genomics

## Abstract

**Background:**

The life-threatening pathogen *Leptospira interrogans* is the most common agent of leptospirosis, an emerging zoonotic disease. However, little is known about the strains that are currently circulating worldwide due to the fastidious nature of the bacteria and the difficulty to isolate cultures. In addition, the paucity of bacteria in blood and other clinical samples has proven to be a considerable challenge for directly genotyping the agent of leptospirosis directly from patient material. Our understanding of the genetic diversity of strains during human infection is therefore limited.

**Methods:**

Here, we carried out hybridization capture followed by Illumina sequencing of the core genome directly from 20 clinical samples that were PCR positive for pathogenic *Leptospira* to elucidate the genetic diversity of currently circulating *Leptospira* strains in mainland France.

**Results:**

Capture with RNA probes covering the *L. interrogans* core genome resulted in a 72 to 13,000-fold increase in pathogen reads relative to standard sequencing without capture. Variant analysis of the genomes sequenced from the biological samples using 273 *Leptospira* reference genomes was then carried out to determine the genotype of the infecting strain. For samples with sufficient coverage (19/20 samples with coverage > 8×), we could unambiguously identify *L. interrogans* serovars Icterohaemorrhagiae and Copenhageni (14 samples), *L. kirschneri* serovar Grippotyphosa (4 samples), and *L. interrogans* serovar Pyrogenes (1 sample) as the infecting strains.

**Conclusions:**

We obtained high-quality genomic data with suitable coverage for confident core genome genotyping of the agent of leptospirosis for most of our clinical samples. The recovery of the genome of the serovars Icterohaemorrhagiae and Copenhageni directly from multiple clinical samples revealed low adaptive diversification of the core genes during human infection. The ability to generate culture-free genomic data opens new opportunities for better understanding of the epidemiology of this fastidious pathogen and pathogenesis of this neglected disease.

**Supplementary Information:**

The online version contains supplementary material available at 10.1186/s12879-023-08126-x.

## Introduction

Leptospirosis is a globally distributed zoonosis responsible for more than one million severe cases and 60,000 deaths per year, with the highest incidence in tropical countries [1]. The agent of leptospirosis belongs to the genus *Leptospira*, which is composed of 68 species and more than 300 serovars [2, 3]. The strains responsible for leptospirosis in humans or animals belong to one of the eight pathogenic *Leptospira* species described to date. Among them, *L. interrogans* is the most frequently encountered worldwide [4] and several studies have shown that strains belonging to the Icterohaemorrhagiae serogroup (*L. interrogans* serogroup Icterohaemorrhagiae), of which the main reservoir is the rat, are responsible for the most severe forms of the disease [5–8].

Pathogenic leptospires are slow-growing bacteria that require a rich culture medium susceptible to contamination by other organisms. Isolation from biological samples is therefore tedious, especially as the bacteria can be present in low concentrations in blood and urine. During the course of infection, the bacteria are present in the blood during the first week after the onset of symptoms, with their concentration determined by qPCR ranging from 10^2^ to 10^6^ *Leptospira*/mL for the peak of leptospiremia [9, 10]. A decreasing number of leptospires are then found in the blood 6–7 days after the onset of symptoms until *Leptospira* nucleic acid is no longer detectable [10, 11]. *Leptospira* can also be detected in urine after symptom onset for a longer duration than blood. However, the bacteria may not be consistently present in the urine during the infection and both their concentration and the duration of their excretion are poorly defined.

The identification of circulating strains in a particular region is essential for establishing appropriate control and prevention measures such as development of vaccines, control of potential reservoirs, information for the general population, etc. Typing of the clinical isolates can also be important for identifying strains or virulence factors associated with disease severity. However, as indicated above, culture isolation is challenging.

Given the value of whole genomes for phylogenetic, epidemiological, and biological studies, there is an increasing interest in obtaining the genomic sequences of pathogens from clinical samples. This is particularly true for pathogens that are found in low quantities in the host organism and difficult to culture, as for pathogenic *Leptospira*. Single alleles, such as *rrs* [12], *ligB* [13], *lfb1* [14], and *secY* [15–17], can be directly amplified from the samples and sequenced for subtyping but this approach provides only low-level resolution and does not allow discrimination between serovars and closely related strains. Multi-locus sequence typing (MLST) schemes using several alleles can also be used for direct typing from clinical samples, but this can result in incomplete allelic profiles [18–20] and they provide limited genetic information on the infecting strain. We recently developed a core genome MLST (cgMLST) scheme based on 545 genes that are highly conserved across the *Leptospira* genus [4]. Our cgMLST scheme allows the identification of pathogenic species, serogroups, and closely related serovars. However, this highly discriminatory approach requires culture isolation of clinical strains. Direct sequencing from clinical samples is hampered by high human host DNA contamination. Illumina sequencing of the cerebrospinal fluid of a patient with neuroleptospirosis showed, for example, only 0.016% of the sequence reads corresponding to the bacterial agent of leptospirosis [21]. Due to the low number of pathogenic microbes in clinical samples, several culture-independent genome sequencing methods have been recently developed using host depletion and/or microbial enrichment approaches. Targeted DNA enrichment, which relies on reference genomes of the target bacteria, has thus been used to retrieve the DNA of bacterial pathogens, such as *Chlamydia trachomatis* [22], *Mycobacterium tuberculosis* [23], and *Treponema pallidum* [24–26], from clinical samples.

Here, we describe a method utilizing biotinylated RNA probes designed specifically for *L. interrogans* DNA to capture the *Leptospira* core genomes defined by our cgMLST scheme [4] directly from routine diagnostic samples. This study demonstrates, for the first time, the successful and accurate high-coverage sequencing of *Leptospira* genomes directly from biological samples.

## Methods

### Samples

Twenty routine diagnostic samples (12 blood, 1 serum, and 7 urine samples) testing positive for Leptospirosis by real-time PCR in the French National Reference Center (NRC, Institut Pasteur) were analyzed in this study (Table 1); there was no attempt of culture isolation for these samples. Total DNA was extracted using DNeasy Blood and Tissue DNA extraction and QIAamp kits (Qiagen) and PCR was performed by real-time PCR using *lfb1* as a target [27]. Sequencing of the PCR products of *lfb1* enabled identification of the *Leptospira* species. *Leptospira interrogans-* or the *L. interrogans*-related species *L. kirschneri*-infected samples with a Ct value ≤ 38 were further selected for this study (Table 1).

**Table 1:** Samples used in this study and identification of the infecting *Leptospira* strain

ID	Source	Patient	Sex	Age	Ct value^a^	Days^b^	Nreads^c^	Bacteria (%)^d^	Species^e^	Serogroup^e^	Serovar^e^	Strain^e^	Reference genome (id)^f^	Number of SNPs
1	Blood	A	M	60	31	Unknown	11,223,500	30	*L. interrogans*	Icterohaemorrhagiae	Copenhageni	L1-130	246	4
2	Urine	B	M	61	30	6	9,523,661	72	*L. interrogans*	Icterohaemorrhagiae	Copenhageni	L1-130	246	4
3	Blood	C	M	41	33	Unknown	11,662,248	20	*L. interrogans*	Icterohaemorrhagiae	Copenhageni	L1-130	246	2
4	Blood	D	M	42	20	2	19,405,989	99	*L. interrogans*	Icterohaemorrhagiae	Copenhageni	L1-130	246	3
5	Blood	E	M	49	34	2	2,817,867	21	*L. interrogans*	Pyrogenes	Zanoni	LT2156	228	1272
6	Serum	F	M	40	34	4	6,936,356	13	*L. kirschneri*	Grippotyphosa	Grippotyphosa	201402975	117	6
7	Blood	G	M	54	27	4	13,037,685	90	*L. kirschneri*	Grippotyphosa	Grippotyphosa	201402975	117	15
8	Blood	H	M	20	32	Unknown	8,739,223	65	*L. interrogans*	Icterohaemorrhagiae	Copenhageni	L1-130	246	4
9	Blood	I	M	69	29	3	12,665,561	65	*L. interrogans*	Icterohaemorrhagiae	Copenhageni	L1-130	246	6
10	Blood	J	M	74	28	0	12,989,647	94	*L. interrogans*	Icterohaemorrhagiae	Copenhageni	L1-130	246	3
11	Blood	K	M	36	32	4	9,589,816	90	*L. interrogans*	Icterohaemorrhagiae	Copenhageni	L1-130	246	5
12	Urine	L	M	24	31	Unknown	10,827,346	65	*L. interrogans*	Icterohaemorrhagiae	Copenhageni	L1-130	246	4
13	Urine	M	M	73	31	5	11,947,042	90	*L. interrogans*	Icterohaemorrhagiae	Copenhageni	L1-130	246	7
14	Urine	N	M	39	35	8	29,832,813	33	*L. interrogans*	Icterohaemorrhagiae	Copenhageni	L1-130	246	3
15	Urine	O	M	59	34	Unknown	12,556,161	29	*L. interrogans*	Icterohaemorrhagiae	Copenhageni	L1-130	246	4
16	Blood	P	M	52	37	3	10,420,132	62	*L. interrogans*	Icterohaemorrhagiae	Copenhageni	L1-130	246	6
17	Blood	Q	M	44	25	5	1,350,830	98	*L. interrogans*	Icterohaemorrhagiae	Copenhageni	L1-130	246	4
18	Blood	R	M	22	38	4	1,0980,69	13	*L. kirschneri*	NA	NA	NA	NA	NA
19	Urine	S	M	33	35	5	1,124,989	35	*L. interrogans*	Icterohaemorrhagiae	Icterohaemorrhagiae	Verdun	106	0
20	Urine	T	M	28	32	4	1,776,330	64	*L. interrogans*	Icterohaemorrhagiae	Copenhageni	L1-130	246	2

*NA* non applicable

^a^Ct value (real-time PCR Lfb1)

^b^Days of sample collection since symptom onset

^c^Number of illumina reads

^d^Proportion of reads from bacteria (%), other reads correspond to human reads

^e^Identification of the infecting strain based on the closest homologue of reference genomes

^f^Identification number (id) of the reference genome in BIGSdb (www.bigsdb.pasteur.fr)

### SureSelect^XT^ target enrichment

In total, 42,117 custom-made SureSelect 120-mer RNA baits (total probe size 1459 Mb) based on 130 *L. interrogans* genome sequences (Additional file 2: Table S1) spanning the 545 core genes previously defined for our cgMLST scheme [4] were designed and synthesized by Agilent Technologies.

For all samples, libraries were prepared following the Sureselect Xt HS Target Enrichment System for Illumina from Agilent Technologies. For pre-capture library preparation, between 2 and 200 ng total gDNA was used. Briefly, samples were mechanically sheared using a Covaris E220, repaired, and the ends A-tailed for barcoded Illumina adapter ligation. Ligated samples were amplified for 14 cycles and library quality was assessed using the Fragment Analyzer HS NGS migration kit. Libraries were captured individually, as recommended by Agilent. Captured libraries were pooled and sequenced using Illumina sequencing technology (Miniseq or Nextseq 500 sequencers). We also sequenced five libraries (samples 3, 4, 5, 7, and 8) before capture on an Illumina Miniseq sequencer to assess the efficiency of the capture method on patient samples.

### Sequence analysis

Prior to mapping, reads were trimmed of the adapters. The variant calling pipeline described herein includes an additional trimming step performed during the mapping step, which removes bases with a Phred score < 30. The estimated depth of coverage was computed using the genome of the *L. interrogans* serovar *Copenhageni* strain Fiocruz L1-130 as a reference. Taxonomic analysis based on Kraken2 [28] using human, bacterial, and viral databases allowed us to classify an average of 99% of the reads with a minimum of 97.2% for one sample.

We generated a database of 273 genome sequences of representative pathogenic *Leptospira* strains from our publicly accessible online genome database https://bigsdb.pasteur.fr/leptospira/, which is based on the software framework Bacterial Isolate Genome Sequence Database [4]. Our allele database includes genomes from eight pathogenic species from 23 serogroups and 59 serovars isolated from patients from 40 countries (Additional file 2: Table S2).

Variant calling analysis was performed using the variant calling pipeline (v0.10.0) from the Sequana project [29] (https://github.com/sequana/variant_calling). Default parameters were used except for the minimum frequency set to 0.5. The mapping step was performed using BWA (v0.7.17) [30] and variant calling was performed using freebayes (v1.3.2) [31]. Annotations were also included in the final HTML report by using prokka [32] annotation of the core genomes. All VCF (variant calling format) files (20 samples times 273 strains) were processed to gather the number of SNPs, INDELs, and MNPs in each sample and each strain using the VCF files and the Python notebooks used to process them are available on Zenodo [https://doi.org/10.5281/zenodo.7584745].

Variants were removed from subsequent analysis if one of the following conditions was met: (i) a frequency of the alternate < 0.5 (minor variants), (ii) a strand balance < 0.2 (or > 0.8), indicating an unbalanced count of forward or reverse reads supporting the variant, or (iii) coverage < 10. For sample 18 (highest Ct), we allowed the depth to be as low as 4.

## Results

Targeted enrichment was applied to 20 biological samples, consisting of blood, serum, and urine samples, from leptospirosis patients (Table 1). *Leptospira* isolates were collected from patients living in mainland France but patient E mentioned having traveled to the Philippines 2 weeks before the onset of symptoms. Among the 20 patients, all were males (71.2%) and the median (range) age was 46 (20–74) years. Patients presented with median (range) of 3.9 (0–8) days of acute illness and most of them exhibited fever, headache and myalgia; (patient Q died).

**Fig. 1:**
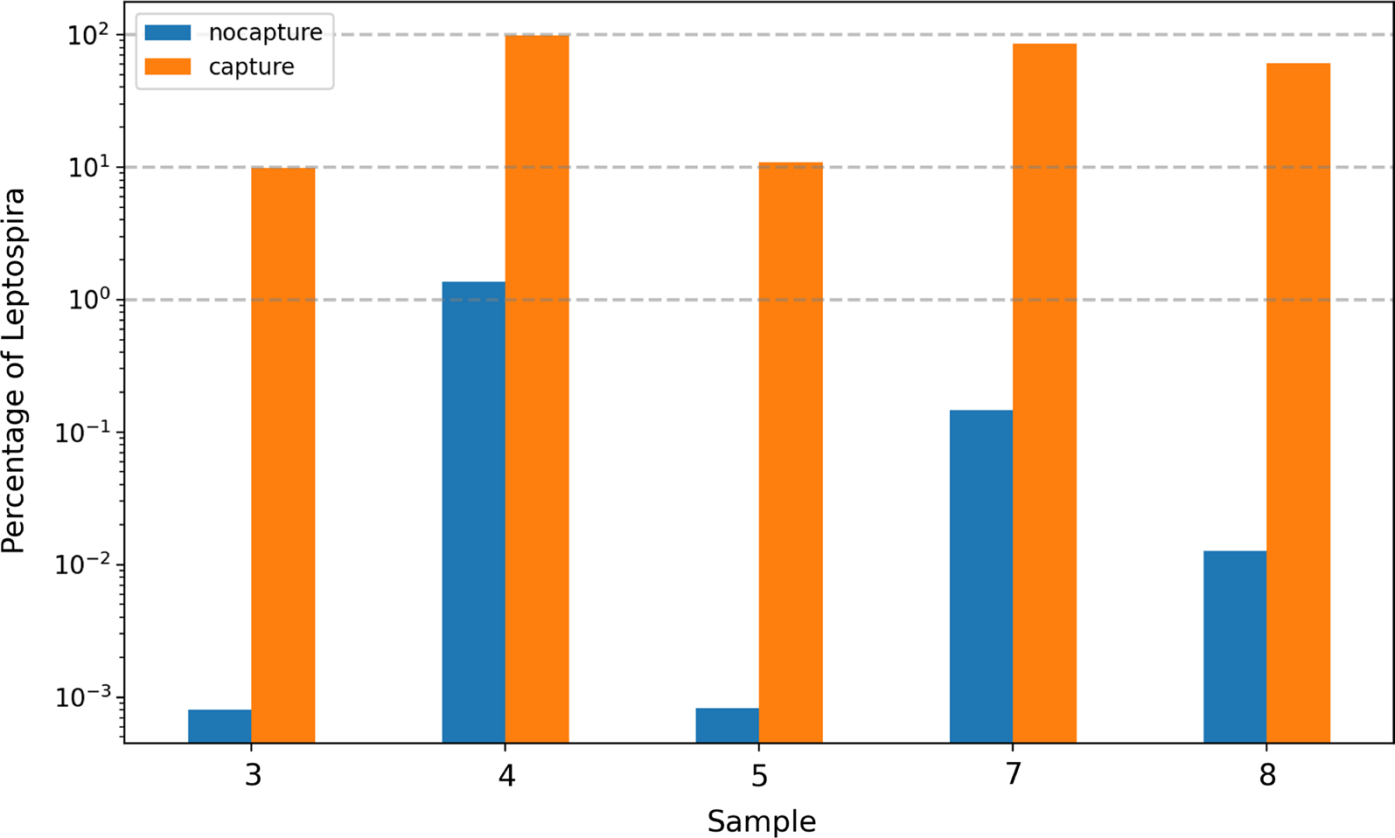
Percentage of *Leptospira* reads found in samples 3, 4, 5, 7, and 8 before (blue bars) and after hybridization capture using* Leptospira* RNA probes (orange bars)

Library preparation, hybridization, and subsequent enrichment were carried out on samples using the SureSelect Target Enrichment System (Agilent Technologies) [33] and custom designed RNA baits. We compared the proportion of reads mapped to the *Leptospira* reference genomes with or without the SureSelect system for five samples to better evaluate the efficiency of Leptospira capture (Fig. 1). The percentages of reads mapped to the *Leptospira* reference genomes for samples prepared without the target-enrichment steps were 0.0008% (sample 3), 1.36% (sample 4), 0.0008% (sample 5), 0.15% (sample 7), and 0.013% (sample 8). The percentages of reads mapped to the Leptospira genomes for the same samples prepared using the SureSelect system jumped to 10%, 98%, 11%, 86%, and 61%, respectively. Thus, capture increased the proportion of Leptospira by several orders of magnitude (72–13,000).

Almost all of the bacterial reads were assigned to the family *Leptospiraceae* (Additional file 1: Fig. S1, S2); viral content was marginal (< 0.1%) (Additional file 1: Fig. S2). The average depth of coverage in the 20 target-enriched samples was 590 ×, ranging from 0.5 × (sample 18) to 6000 × (sample 4) (Additional file 1: Fig. S3), hence leading to a large standard deviation of 1320. Coverage across the genomes was computed using the Sequana coverage tool [34] to more precisely characterize the genomic variations in the different samples (Additional file 1: Fig. S4). The enrichment results, together with the average coverage (Additional file 1: Fig. S3), highlights several key points. Most samples had coverage above 50 ×, except samples 3, 5, 6, and 18. The coverage of samples 3 and 5 was still sufficient, with 42 × and 28 ×, respectively. Sample 6 had a low coverage of 8 ×. Finally, sample 18 was more problematic, as its coverage was below 1 ×. It was also possible to assess the breadth of coverage (percentage of bases covered by at least one read) using *L. interrogans* serogroup serovar Copengageni (id246) as a reference (Additional file 1: Fig. S4); it was above 99.5% for most samples, except for sample 6, which had a breadth of coverage of 85%, and sample 18, for which the coverage was only about 3%.

**Fig. 2:**
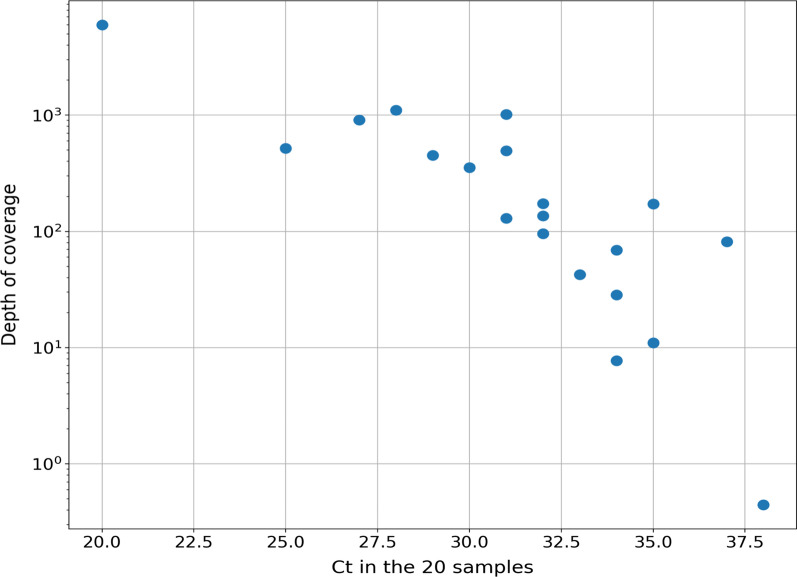
Final depth of coverage as a function of the measured Ct for the 20 samples. The depth of coverage is based on the *L. interrogans* core genome. The correlation coefficient between the depth of coverage and Ct values was 0.76

The Ct values of real-time PCR targeting the pathogen-specific target lfb1 ranged between 20 and 38, corresponding to 105 bacteria/µl to less than 1 bacterium/µl [27]. There was a good correlation between the Ct values and the proportion of mapped *Leptospira* reads and depth coverage (Fig. 2). Thus, the six samples with more than 90% *Leptospira* reads (samples 4, 7, 10, 11, 13, and 17) had Ct values < 32 (Table 1).

**Fig. 3:**
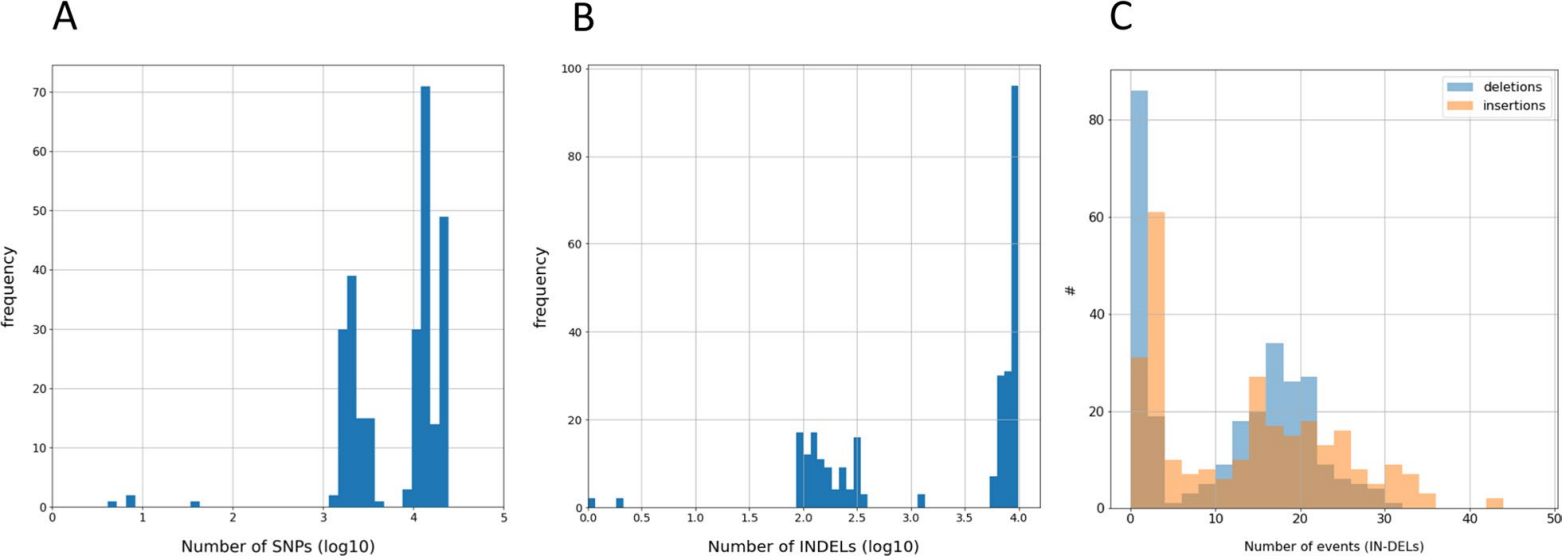
Analysis of multiple nucleotide polymorphisms. Histograms of the number of single-nucleotide polymorphisms (SNPs) (**A**), INDELs, including multiple nucleotide polymorphisms (MNPs) (**B**), and insertions and deletions, excluding MNPs (**C**) found in sample 1 across all 273 *Leptospira* genomes

The variant calling approach was performed to identify the genotype of each sample. We searched for the closest genome for a given sample using a database of 273 genomes of pathogenic *Leptospira* strains from different species, serogroups, and serovars originating from various geographic areas (Additional file 2: Table S2) by minimizing the distance between the raw sequencing data of the sample and the *Leptospira* reference genomes. The distance used was the count of high-quality variants found in a given sample relative to the different strains, as explained below. As the capture was designed using probes covering the core genomes only, we solely considered the core genome of the 273 strains; the average core genome length was 574.7 ± 8.5 kb. Although coverage was uneven in some samples, with the presence of spikes (excess coverage in short regions, low frequency trend in sample 20) (Additional file 1: Fig. S4), it was generally high enough for variant calling analysis. We first examined the number of SNPs. The distribution of SNPs across all genomes and samples was highly variable, with values ranging from 0 to 23,000 SNPs (average of 10,000). The SNP count histogram across the 273 strains is shown in Additional file 1: Fig. S5, in which 95% of the strains show a count above 100, whereas a few strains had SNP counts below 10 (Fig. 3A; Additional file 1: Fig. S5).

**Fig. 4:**
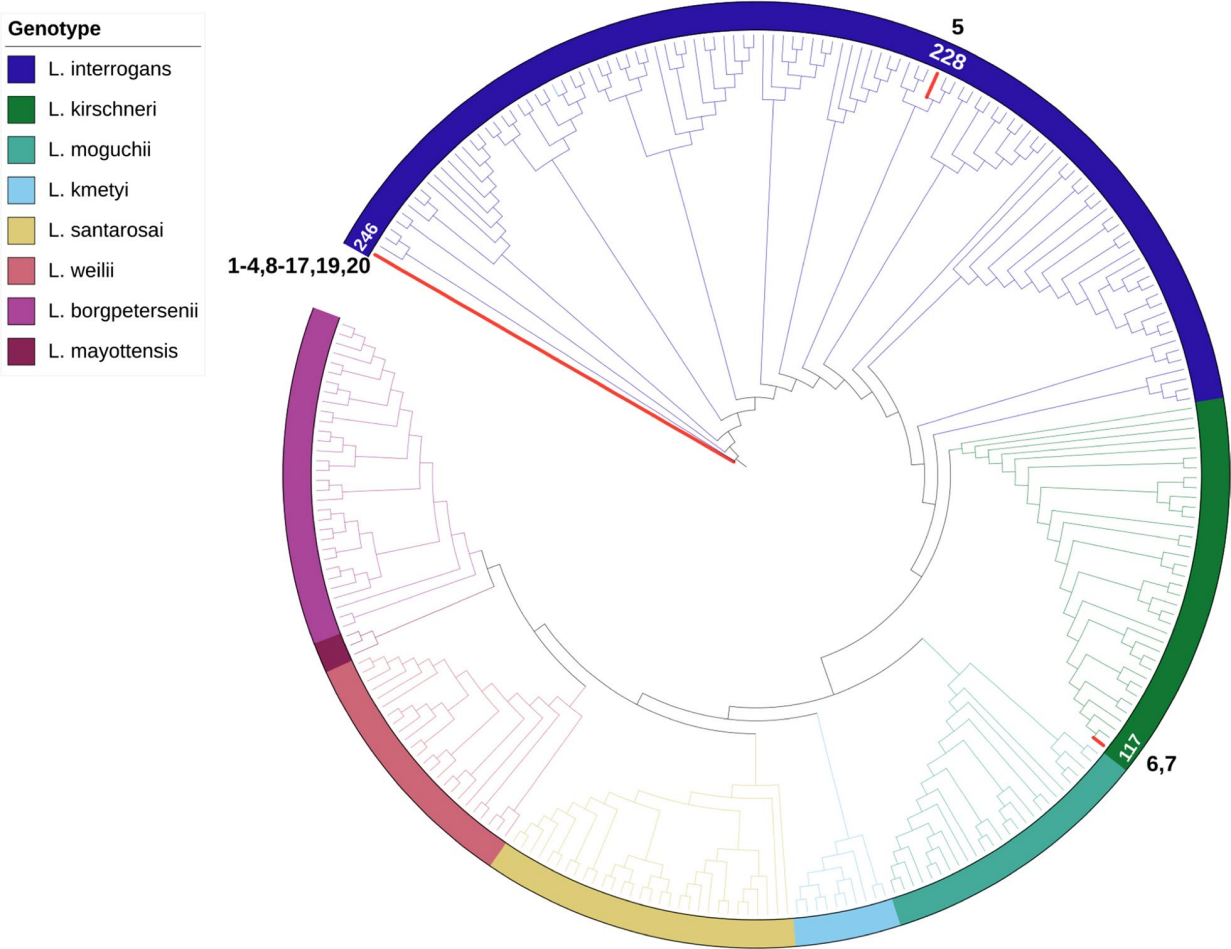
Phylogeny of *Leptospira* genomes and the sequenced samples. The *Leptospira* sequences from samples were compared using a database of 273 core genomes of pathogenic *Leptospira* strains (Additional file 1: Table S2) to find the closest homologue. Samples 1–4, 8–17, 19, 20 had a minimal number of single nucleotide polymorphisms (SNPs), deletions, insertions, and multiple nucleotide polymorphisms (MNPs) relative to id246 (*L. interrogans* sv Copenhageni). Samples 6 and 7 are closely related to id117 (*L. kirschneri* sv Grippotyphosa) and Sample 5 is closely related to id228 (*L. interrogans* sv Pyrogenes). The phylogenetic tree was generated using multiple alignment (mafft v7.490) and RaxML software to infer the underlying phylogenetic tree. Visualization was performed via the iTOL web service (https://itol.embl.de)

Interestingly, most of the examined samples had less than 10 SNPs relative to the reference genomes of *L. interrogans* serovar (sv) Icterohaemorrhagiae strain RGA (id97), *L. interrogans* sv Icterohaemorrhagiae strain Verdun (id106), and *L. interrogans* sv Copenhageni strain Fiocruz L1-130 (id246) (Table 2). These three strains are phylogenetically related (Fig. 4) and belong to *L. interrogans* serogroup (sg) Icterohaemorrhagiae. In particular*, L. interrogans* sv Copenhageni (id246) appeared to be the strain with the smallest number of SNPs in most samples (if we ignore samples 5, 6, and 7 that were distant from the serogroup Icterohaemorrhagiae, and sample 18, which had no SNPs due to low coverage). Using the minimum number of SNPs as the criteria for assignation, 15 of the 19 samples were assigned to *L. interrogans* sv Copenhageni (id246). Sample 19 had 2 SNPs in id246 and none in id106 and was thus assigned to *L. interrogans* sv Icterohaemorrhagiae (id106). Sample 5 was assigned to *L. interrogans* serovar Zanoni (id228) from serogroup Pyrogenes with large number of SNPs (1272); the second best hit among the 273 reference genomes was another Pyrogenes strain (id11). Samples 6 and 7 were assigned to *L. kirschneri* sv Grippotyphosa (id117) with 6 and 15 SNPs respectively (Table 2). Sample 18 was excluded from the analysis due to a low average. Nevertheless, if we decreased the required depth for variant calling to 4 × then the number of SNPs rose to approximately 20, on average, across all genomes (Additional file 1: Fig. S5). One had no SNPs (*L. kirschneri* sg Grippotyphosa; id149), although 15 other strains had 1 or 2 SNPs. These strains are close to each other in the phylogenetic tree (Fig. 4) and belong to *L. kirschneri*. In particular, id117 has only 1 SNP, and was also the best hit for samples 6 and 7 (Table 2).

**Table 2:**
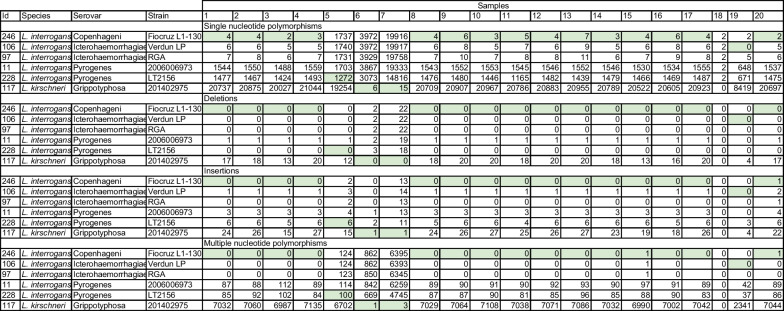
SNPs, INDELs, and MNPs found in the 20 samples

The *Leptospira* sequences from samples were compared using a database of 273 core genomes of pathogenic *Leptospira* strains to find the closest homologue

To confirm these results, we also examined other types of variants: insertions, deletions, and multiple nucleotide polymorphisms (MNPs). The counts of insertions and deletions are hereafter summarized together as the number of INDELs. The number of INDELs in sample 1 varied from 0 up to 10,000 (Fig. 3B, C). Using the minimum of the total count of INDELs and MNPs across genomes, we found results similar to those found with the SNPs analysis. Sample 6 had several best hits: id117, id110, and id700 that led to no deletions, one insertion and one MNP each. Interestingly, id117, id110, id700 belong to serogroup Grippotyphosa and they are next to each other in the phylogenetic branch. For sample 7, id117 was the closest with one insertion and 2 MNPs. Other reference genomes from serogroup Grippotyphosa (id110, id315, and id700) were closeby with only 1 or 2 additional INDELs/MNPs. For sample 5, id228 had the minimum number of INDELs/MNPs with 0 deletions, 6 insertions and 100 MNPs. All other samples, which were close to id246 in terms of SNPs, had no INDELs or MNPs in id246, id97 and id106 (except sample 15, which had 1 MNP and sample 20 with several insertions or MNPs) (see Table 2 for details). Overall, the studies of SNPs, INDELs and MNPs converge to provide a robust assignation for each sample.

We further analyzed the SNPs and INDELs identified in coding regions for samples 1–4, 8–17 and 19–20 which were assigned to *L. interrogans* serovars Copenhageni and Icterohaemorrhagiae (Tables 1, 2). Comparison of genome of *L. interrogans* serovar Copenhageni strain Fiocruz L1-130 with the sequences of the 545 core genes of the samples resulted in the identification of 2 to 7 SNPs which were distributed in 22 different genes (Table 3). SNPs in genes LIC11311, LIC13481 and LIC12955 were conserved in most if not all samples (Table 3); other SNPs were sample specific. Of the identified mutations in the core genes, 9 were synonymous and 13 were non-synonymous. The vast majority of genes with non-synonymous SNPs are annotated as involved in various biological processes such as amino acid transport and metabolism, energy production and conversion, lipid transport and metabolism, transcription, translation, ribosomal structure and biogenesis, etc. (Additional file 2: Table S3). We also noted 1 insertion causing a frameshift in LIC11604 which encodes a hypothetical protein (Additional file 2: Table S3).

**Table 3:** Genes showing SNPs and INDELs

	Genes showing mutations									
	LIC10121	LIC10339	LIC10484	LIC10580	LIC10842	LIC10874	LIC12841	LIC11070	LIC11265	LIC11311
Sample	*rsgA*	*clpC*	*thrC*	*msbA*	*dapA*	*RS04520*	*dapL*	*dhaT*		
1										623
2										623
3										623
4										623
8										623
9			1123	629						623
10										623
11	372									623
12										623
13		2207				293	220			623
14										623
15										623
16								930	107	623
17					902					623
19										623
20										623

	Genes showing mutations											
	LIC11335	LIC11374	LIC12764	LIC11455	LIC11604	LIC11518	LIC11570	LIC11791	LIC12870	LIC12955	LIC10974	LIC13481
Sample	*groL*	*fliR*	*ilvB*	*mfd*			*pulD*	*recG*	*rplB*	*fruA*	*fadA*	*ycfH*
1		12								30		721
2			666							30		721
3												721
4										30		721
8								1373		30		721
9							1819			30		721
10										30		721
11										30	549	721
12				1984						30		721
13						1928				30		721
14										30		721
15									704	30		721
16	1586									30		721
17										30		721
19												721
20					675							721

The genome of *L. interrogans* serovar Copenhageni strain Fiocruz L1-130 (id246) was compared with the sequences from samples which were assigned to *L. interrogans* serovars Copenhageni and Icterohaemorrhagiae (Tables 1, 2). The position of mutations is indicated. More informations on the identified mutations can be found in Additional file 1: Table S3.

## Discussion

Genomics studies are proving to be important for the characterization of pathogen diversity and pathogenicity, yet the fastidious growth of *Leptospira* and the low abundance of *Leptospira* in clinical samples has presented a challenge for such studies. Thus, it can take up to four months of incubation for a primary culture to become positive [35, 36]. In addition, certain *Leptospira* serovars require additional culture media supplements for their growth [37]. Our data demonstrate, for the first time, the suitability of target-capture technology for purifying very low quantities of *Leptospira* DNA from complex DNA populations in which the host genome is in vast excess. We show the successful enrichment of *Leptospira* DNA by the significant increase in the ratio of bacteria:human DNA post-hybridization in a subset of samples. The Ct strongly correlated with capture efficiency. In six samples with a high leptospiral burden (qPCR Cts between 20 and 31 or 105 bacteria/µl to approximately 102 bacteria/µl), *Leptospira* reads accounted for > 90% of the total reads. Targeted enrichment was applied to blood, serum, and urine samples from leptospirosis patients a few days after symptom onset (0–8 days, average of 3.9 days), showing that the method can be applied to routine diagnostic samples. We show that enrichment of *L. interrogans* reads provides sequencing data that match the quality and quantity of data obtained via sequencing from cultures, with coverage above 50 × for 16 of the 20 samples, providing an opportunity to compare *Leptospira* strains from routine diagnostic samples with greater resolution than previously possible. Today, this approach is still relatively expensive, currently costing approximately $300 per sample in our laboratory, but as next-generation sequencing costs continue to decline, this approach should become more affordable and accessible. To reduce the cost in future studies, samples can be barcoded and pooled before enrichment, thus enabling multiplexing of hybridization reactions. This approach was already been proven to considerably decrease the cost [24, 38].

The use of specific probes for *L. interrogans* is justified by the cosmopolitan nature of this species, which is found worldwide [4]. The species *L. interrogans* also hosts the most pathogenic serovars, such as those belonging to the Icterohaemorrhagiae serogroup [5–8]. Finally*, L. interrogans* is particularly appropriate for the use of target enrichment, as it has a relatively well-characterized clonal nature and *L. interrogans* strains from different origins show high genetic relatedness [4]. The specificity of the target enrichment probe sets was confirmed by our ability to specifically target *L. interrogans* (17/20). Furthermore, we were also able to target *L. kirschneri* (3/20), which is the closest species phylogenetically to *L. interrogans* (Table 1).

More importantly, this enrichment method effectively captures regions of diversity in the *Leptospira* core genome, which enables precise molecular typing of infecting strains. Comparison of these assembled sequences to the pathogenic *Leptospira* reference core genomes revealed only a limited number of SNPs for most of the samples. Remarkably, the number of SNPs for most samples was as low as a few (e.g., 4 SNPs in sample 1 on strain Fiocruz L1-130), even for samples with a low sequencing yield. Analyzing the SNPs, INDELs, or MNPs independently also provided coherent results, leading to robust assignation. There is however an exception with sample 5 which presents more than 1,200 SNPs with the closest reference genome which belongs to serogroup Pyrogenes. Interestingly this sample was collected from a patient who travelled in the Philippines where Pyrogenes is the most prevalent serogroup in both humans and animals [18]. However, the high number of SNPs reported for this sample suggests that the genome of the infecting strain is not present in our database. Further work will need to isolate and sequence more strains from patients to provide a better picture of the strains that are circulating worldwide.

Because we have a significant number of samples from patients infected with *L. interrogans* serovars Copenhageni and Icterohaemorrhagiae, we can analyze the genetic diversity of the core genes among these samples. Direct sequencing from clinical samples allows to get rid of the numerous mutations that occur during in vitro passages as previously described [39–42]. Previous comparative genomic studies of in vitro cultures from *L. interrogans* serovars Copenhageni and Icterohaemorrhagiae from different origin revealed that the genomes of these two serovars are highly conserved [43]. Similarly, we found a low proportion of mutations among the core genes during human infection and we did not identify any bias toward any particular biological function. One of the limitations of our study is that we analyze only a part of the genome (575/4600 kb or 12.5%) and we don’t have access to the accessory genome which usually includes a number of virulence functions. In the near future, custom synthesized RNA probe sets could be designed to span the entire chromosome of *L. interrogans*. This will provide insights on the bias that may be introduced by culture, as previously shown for the spirochete *Treponema pallidum* [44], as well as increase our understanding of genetic diversity among strains and its impact on immune evasion, persistence and disease outcome.

## Supplementary Information

**Additional file 1: Fig. S1**. Proportion of reads of the 20 samples assigned to bacteria genomes (blue bars) and proportion of reads assigned specifically to the Leptospiraceae family (orange bars). **Fig S2** Taxonomic assignation of the reads of the 20 samples by Kraken2. **Fig. S3** Coverage depth of the L. interrogans core genome across samples. The coverage depth on average for all samples is above 50X (top dashed red line) in most samples (sample 3 coverage equal 42X). Sample 5 and 6 have low coverage of 28X and 8X. Sample 18 coverage is below 1X. Black bars give the ±1 standard deviation. **Fig. S4** Depth coverage of the 20 samples across the L. interrogans core genome. **Fig. S5** Histogram of SNP counts found in the 20 samples across all 273 genomes. For each of the 20 samples, we called variants using the 273 references of Leptospira (Additional file 2: Table S2) independently. For each genome, we obtained a set of SNPs that were filtered out to remove low quality variants (frequency below 10, uneven strand balance). Reference genomes distant from a sample led to thousands of SNPs while closely-related genomes led to less than 10 SNPs. Minimizing the count gives the closet genome to a given sample. Number of SNPs found in the 20 samples across all 273 genomes. Most genomes have more than 10,000 SNPs while only a few exhibit SNPs below 100.

**Additional file 2: Table S1** *L. interrogans* strains used for the design of probes. **Table S2** Database of Leptospira core genomes used for variant calling. **Table S3** Genes showing SNPs and INDELs. The genome of L. interrogans serovar Copenhageni strain Fiocruz L1-130 (id246) was compared with the sequences from samples which were assigned to *L. interrogans* serovars Copenhageni and Icterohaemorrhagiae (Tables 1, 2)

## Data Availability

The raw sequencing data have been deposited in array express at EMBL-EBI (https://www.ebi.ac.uk/) with the accession number E-MTAB-11667.
