# Supplementary material for: Core genome sequencing and genotyping of Leptospira interrogans in clinical samples by target capture sequencing
Source: BMC Infect Dis. 2023 Mar 14;23:157. doi: 10.1186/s12879-023-08126-x (PMC10012794; doi:10.1186/s12879-023-08126-x)
Supplement: Supplementary file 1 — Additional file 1: Fig. S1. Proportion of reads of the 20 samples assigned to bacteria genomes (blue bars) and proportion of reads assigned specifically to the Leptospiraceae family (orange bars). Fig S2 Taxonomic assignation of the reads of the 20 samples by Kraken2. Fig. S3 Coverage depth of the L. interrogans core genome across samples. The coverage depth on average for all samples is above 50X (top dashed red line) in most samples (sample 3 coverage equal 42X). Sample 5 and 6 have low coverage of 28X and 8X. Sample 18 coverage is below 1X. Black bars give the ±1 standard deviation. Fig. S4 Depth coverage of the 20 samples across the L. interrogans core genome. Fig. S5 Histogram of SNP counts found in the 20 samples across all 273 genomes. For each of the 20 samples, we called variants using the 273 references of Leptospira (Additional file 2: Table S2) independently. For each genome, we obtained a set of SNPs that were filtered out to remove low quality variants (frequency below 10, uneven strand balance). Reference genomes distant from a sample led to thousands of SNPs while closely-related genomes led to less than 10 SNPs. Minimizing the count gives the closet genome to a given sample. Number of SNPs found in the 20 samples across all 273 genomes. Most genomes have more than 10,000 SNPs while only a few exhibit SNPs below 100. [file 12879_2023_8126_MOESM1_ESM.pdf]

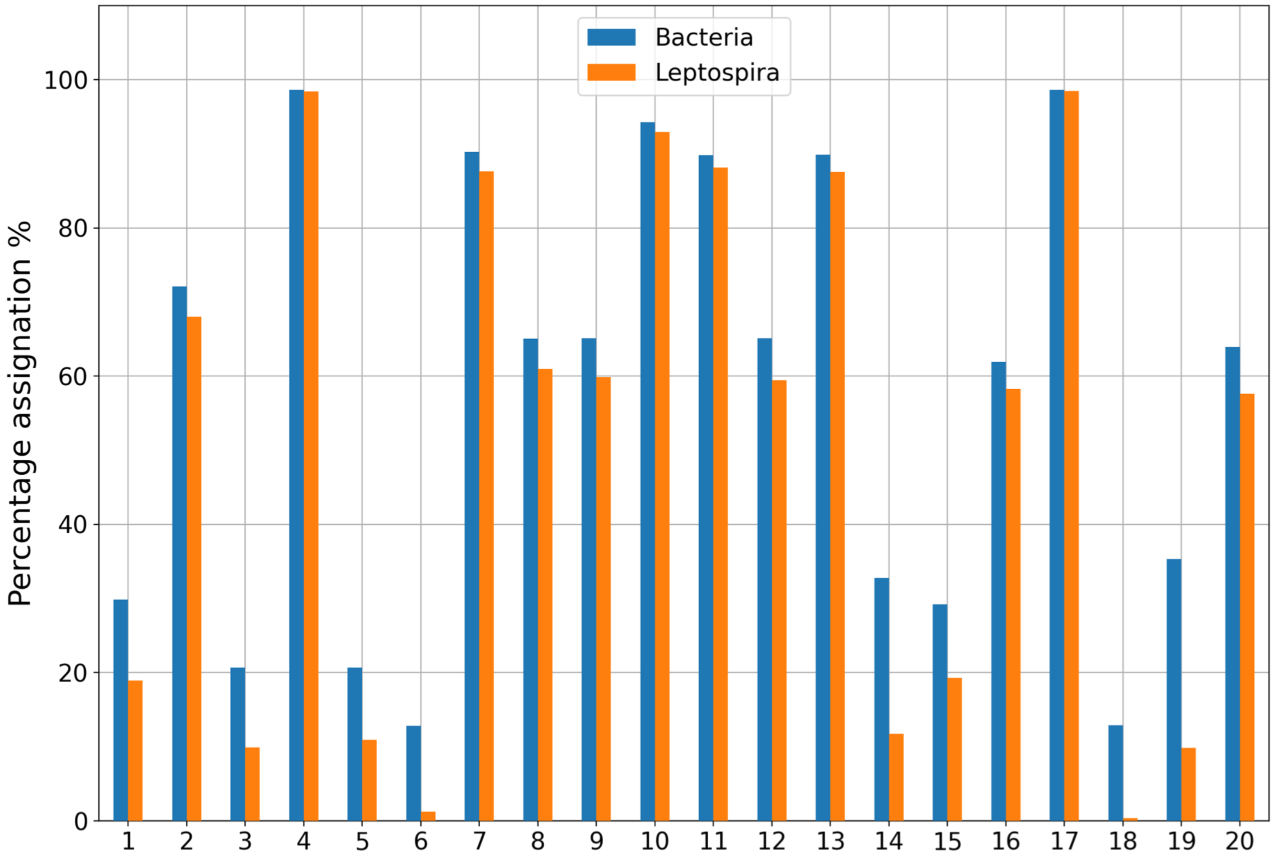

Sup. Fig. 1 : Proportion of reads of the 20 samples assigned to bacteria genomes (blue bars) and proportion of reads assigned specifically to the *Leptospiraceae* family (orange bars).

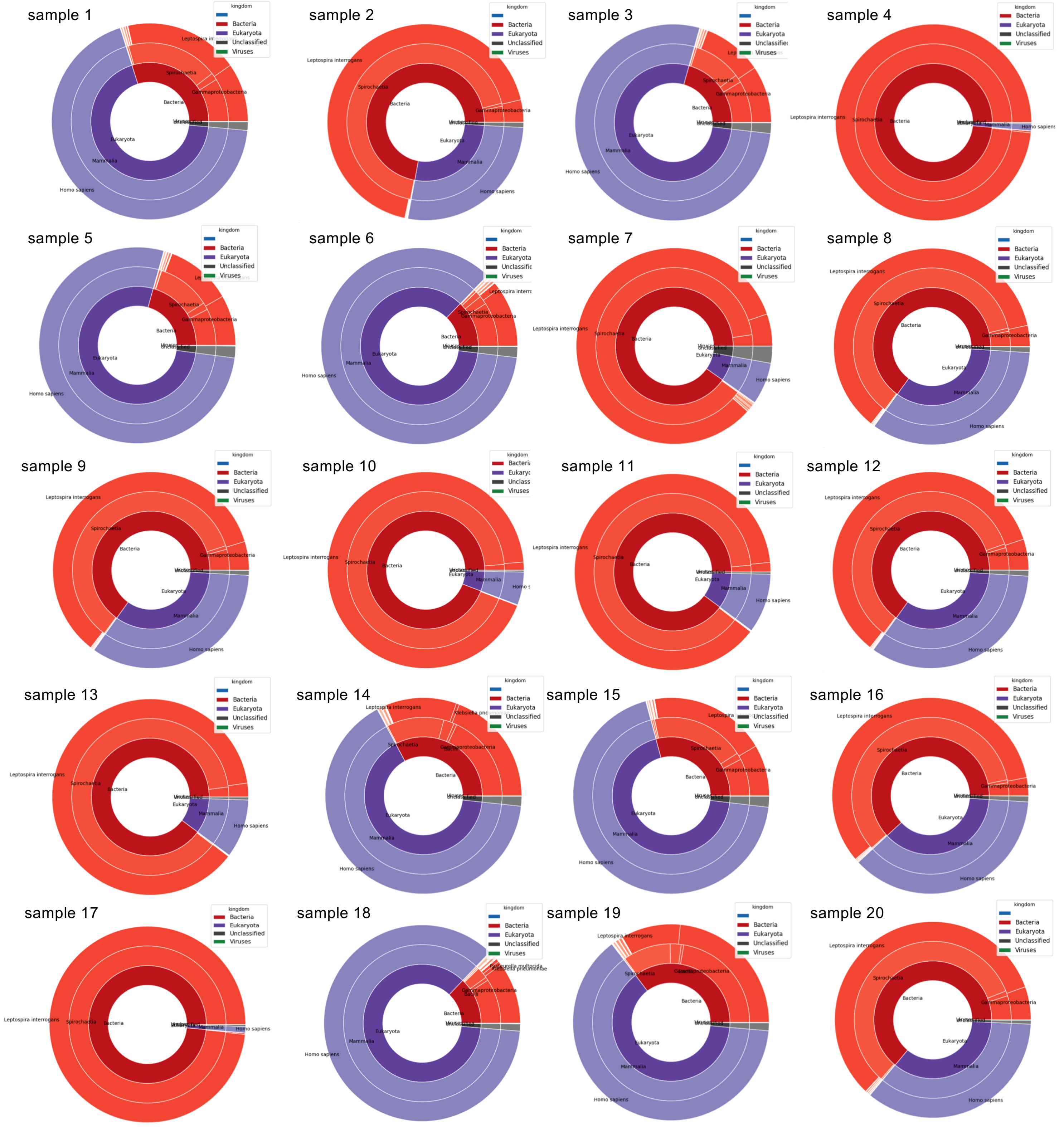

Sup. Fig 2 : Taxonomic assignation of the reads of the 20 samples by Kraken2

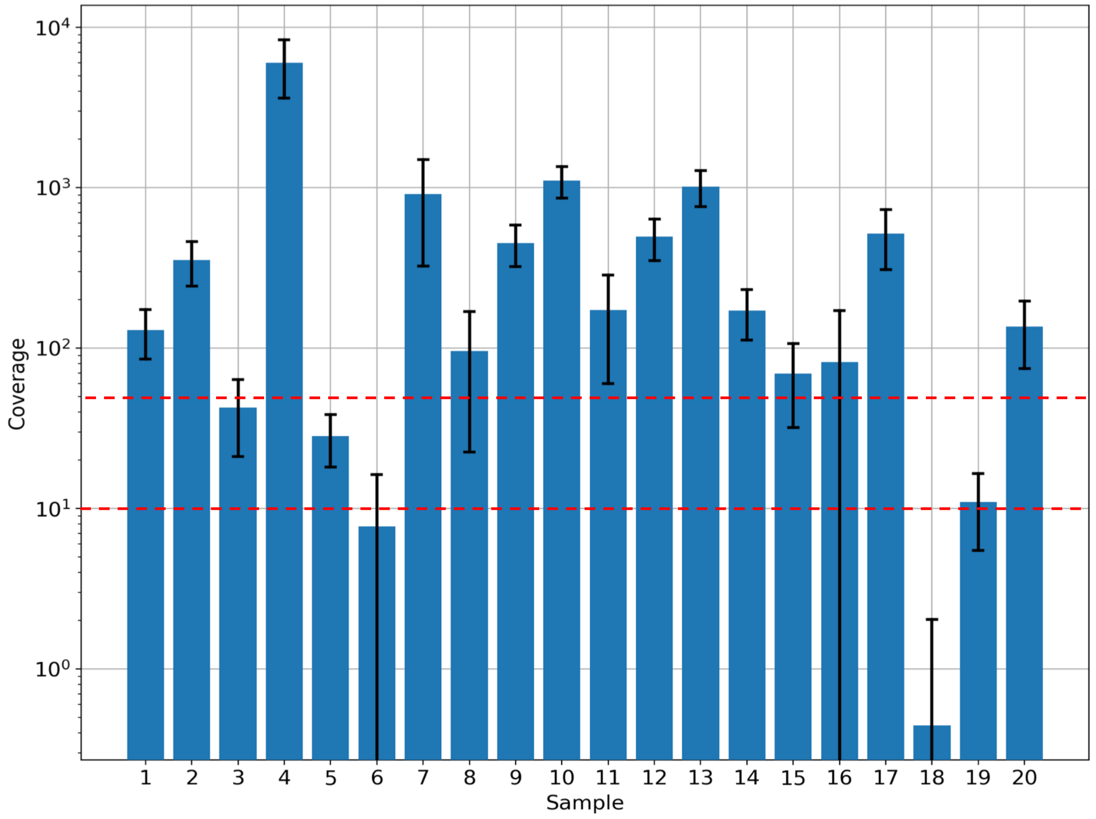

Sup. Fig. 3 : Coverage depth of the *L. interrogans core* genome across samples. The coverage depth on average for all samples is above 50X (top dashed red line) in most samples (sample 3 coverage equal 42X). Sample 5 and 6 have low coverage of 28X and 8X. Sample 18 coverage is below 1X. Black bars give the ±1 standard deviation.

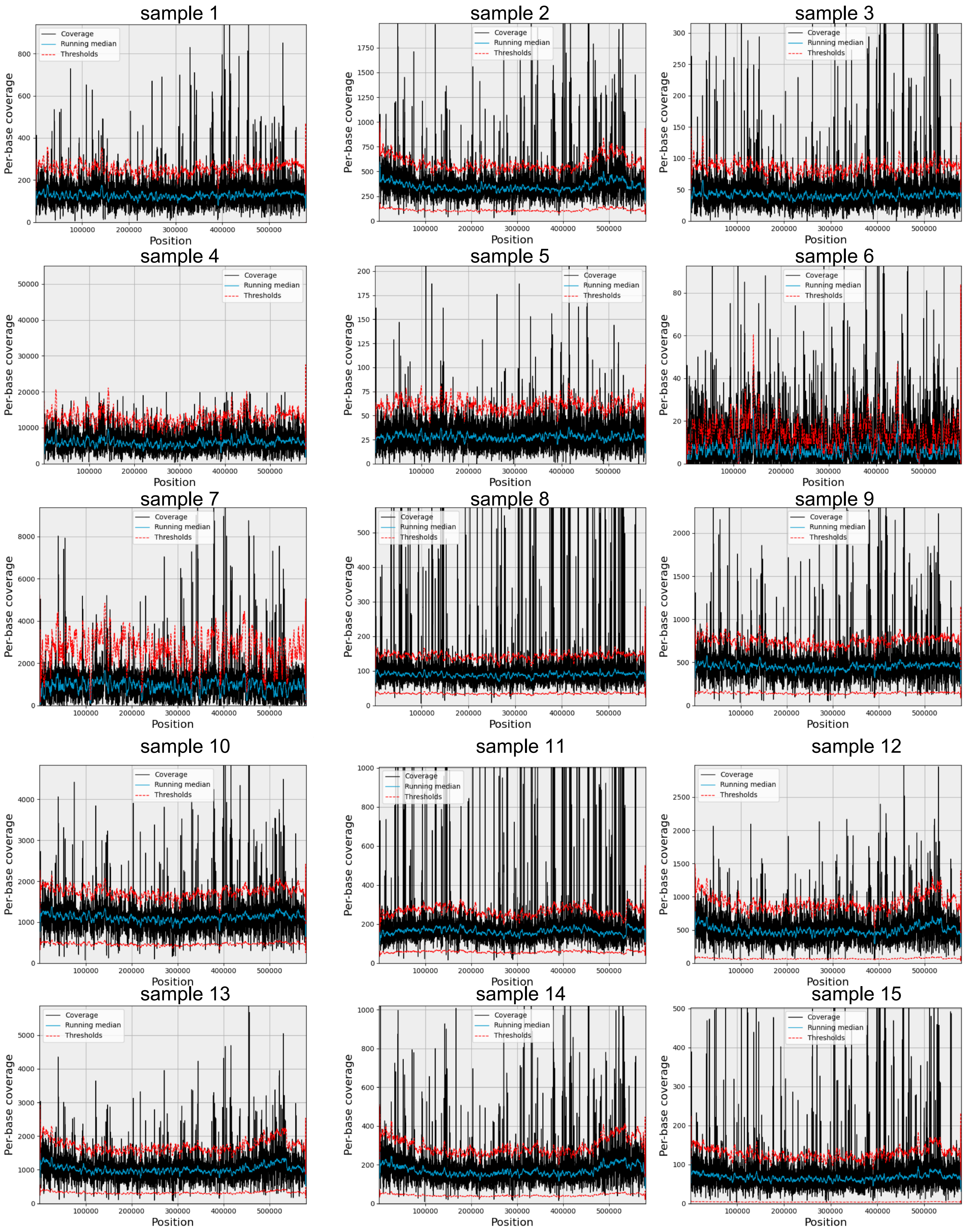
sample 1
sample 2
sample 3
sample 4
sample 5
sample 6
sample 7
sample 8
sample 9
sample 10
sample 11
sample 12
sample 13
sample 14
sample 15
Coverage
Running median
Thresholds
Per-base coverage
Position

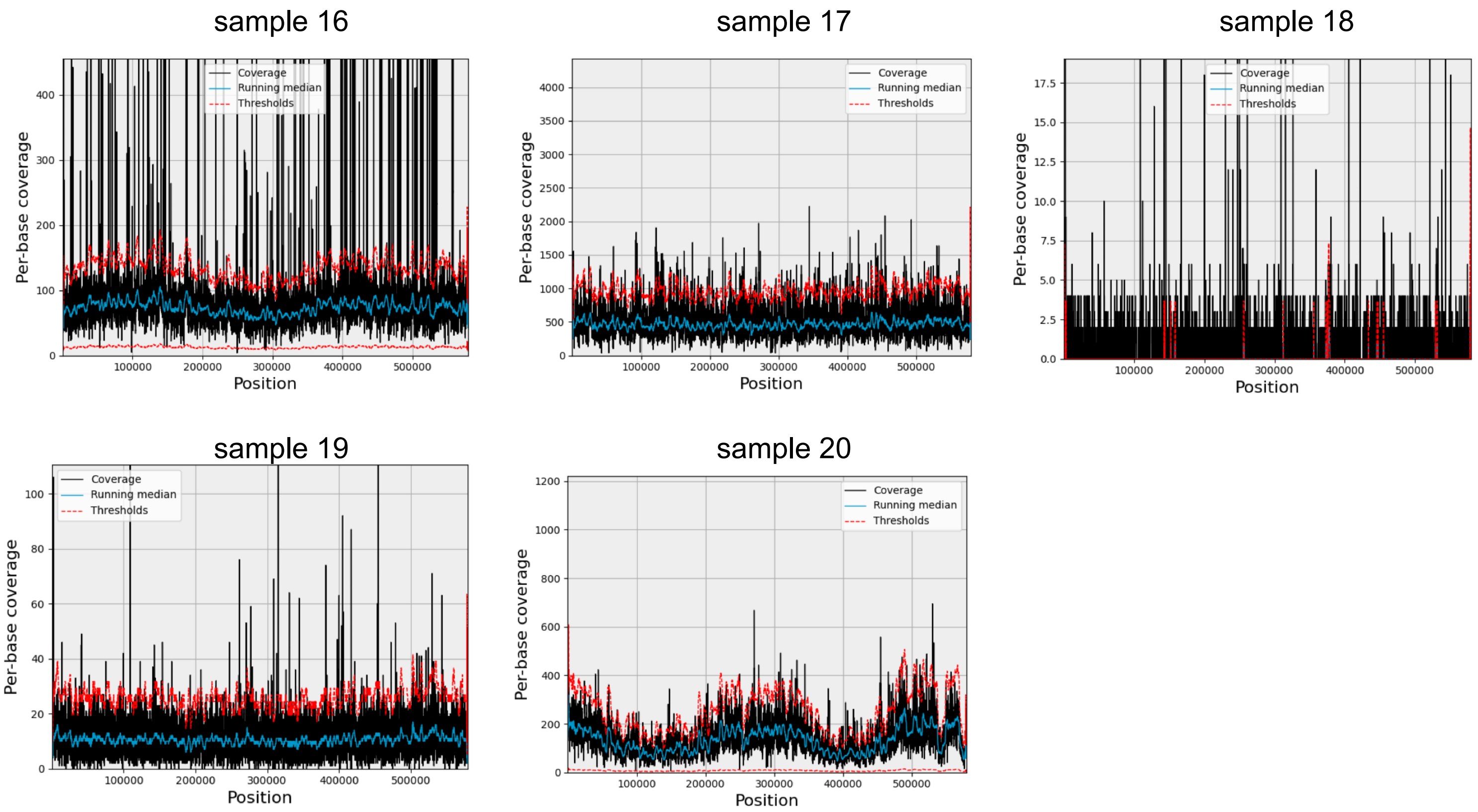

Sup. Fig. 4 : Depth coverage of the 20 samples across the *L. interrogans* core genome

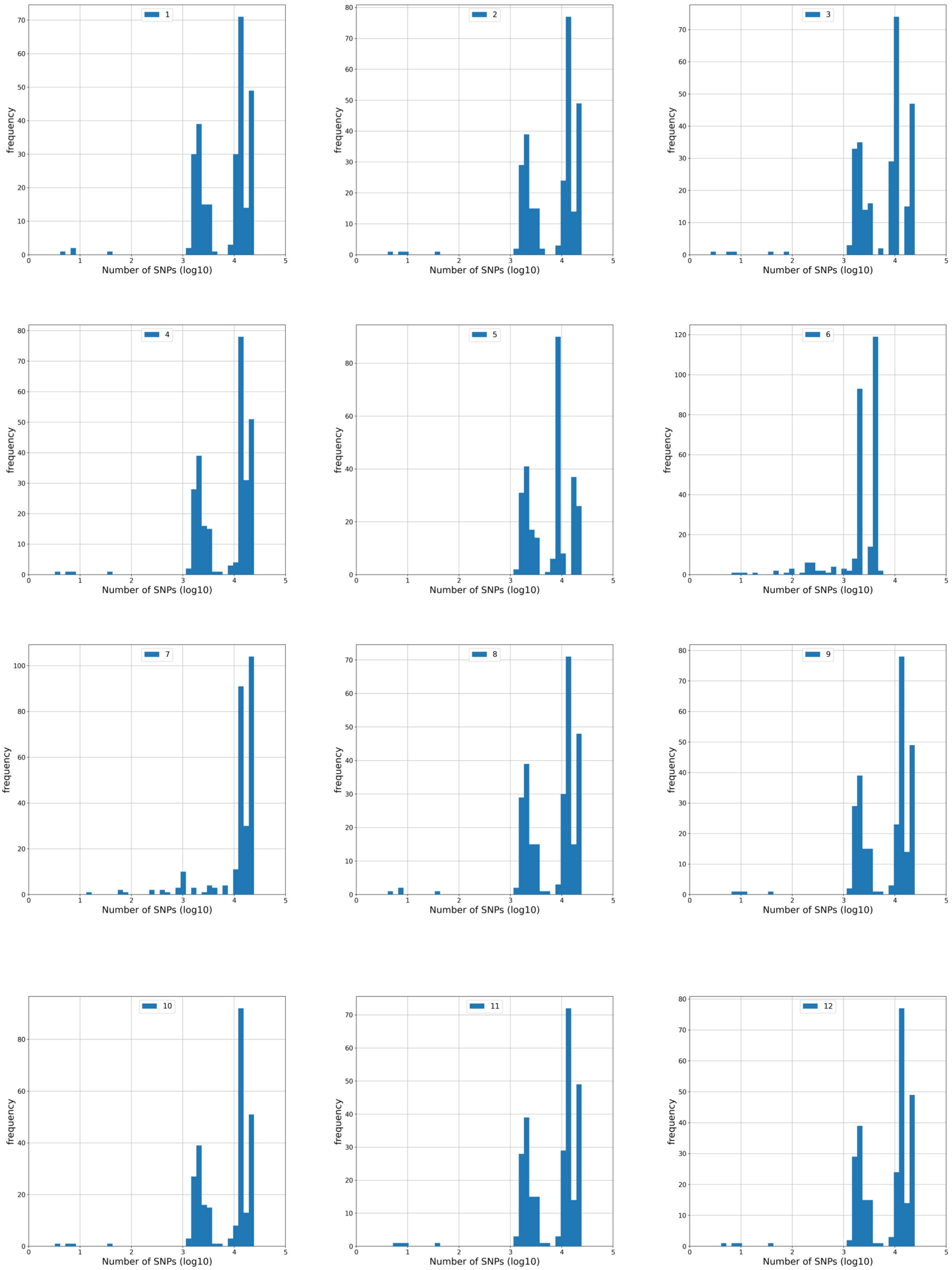
1
2
3
4
5
6
7
8
9
10
11
12
frequency
Number of SNPs (log10)

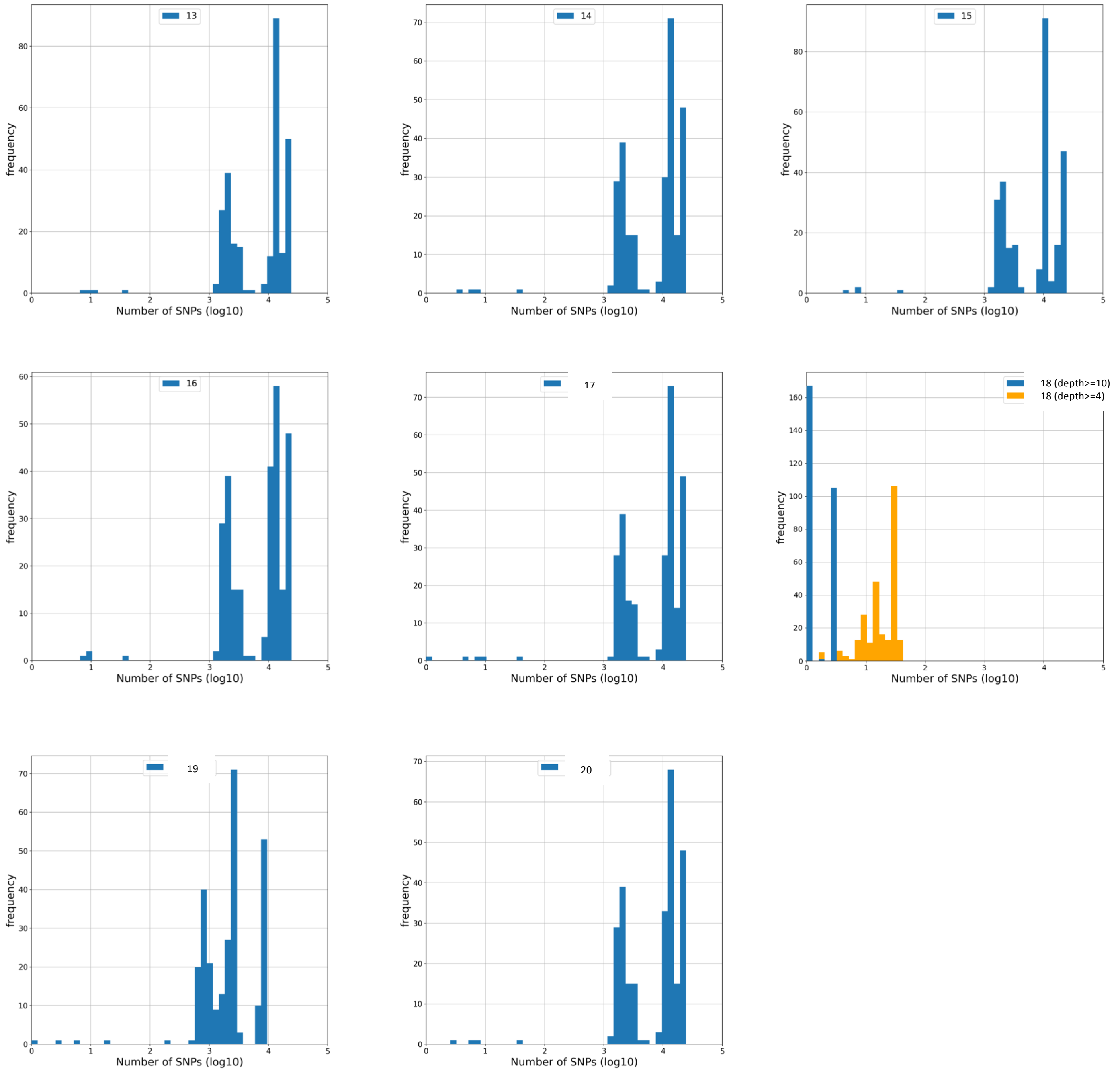

Sup. Fig. 5 : Histogram of SNP counts found in the 20 samples across all 273 genomes.

For each of the 20 samples, we called variants using the 273 references of *Leptospira* (sup. Table 2) independently. For each genome, we obtained a set of SNPs that were filtered out to remove low quality variants (frequency below 10, uneven strand balance). Reference genomes distant from a sample led to thousands of SNPs while closely-related genomes led to less than 10 SNPs. Minimizing the count gives the closet genome to a given sample. Number of SNPs found in the 20 samples across all 273 genomes. Most genomes have more than 10,000 SNPs while only a few exhibit SNPs below 100.
