# Supplementary material for: Core genome sequencing and genotyping of Leptospira interrogans in clinical samples by target capture sequencing
Source: BMC Infect Dis. 2023 Mar 14;23:157. doi: 10.1186/s12879-023-08126-x (PMC10012794; doi:10.1186/s12879-023-08126-x)
Supplement: Supplementary file 2 — Additional file 2: Table S1 L. interrogans strains used for the design of probes. Table S2 Database of Leptospira core genomes used for variant calling. Table S3 Genes showing SNPs and INDELs. The genome of L. interrogans serovar Copenhageni strain Fiocruz L1-130 (id246) was compared with the sequences from samples which were assigned to L. interrogans serovars Copenhageni and Icterohaemorrhagiae (Tables 1, 2) [file 12879_2023_8126_MOESM2_ESM.pdf]

Supplementary Table 1 : *L. interrogans* strains used for the design of probes

id [1]	isolate	species	serovar	serogroup	country	CG [2]
3	2006006986	interrogans	Grippotyphosa	Grippotyphosa	Egypt	2
10	2006006972	interrogans	Copenhageni	Icterohaemorrhagiae	Egypt	6
11	2006006973	interrogans	Pyrogenes	Pyrogenes	Egypt	3
12	2006006976	interrogans	Bataviae	Bataviae	Egypt	4
15	Sri Lanka 30	interrogans	Pyrogenes	Pyrogenes	Sri Lanka	9
18	TE 1992	interrogans	Lora	Australis	Tanzania	12
24	IPAV	interrogans	Lai	Icterohaemorrhagiae	China	16
25	56609	interrogans	Linhai	Grippotyphosa	China	17
26	Lai	interrogans	Lai	Icterohaemorrhagiae	China	16
27	56601	interrogans	Lai	Icterohaemorrhagiae	China	16
31	Hardjoprajitno	interrogans	Hardjo	Sejroe	Indonesia	19
32	Kariadi-Satu	interrogans	Bataviae	Bataviae	Indonesia	4
33	Swart	interrogans	Bataviae	Bataviae	Indonesia	4
37	UP-MMC-NIID HP	interrogans	Manilae	Pyrogenes	Japan	23
38	UP-MMC-NIID LP	interrogans	Manilae	Pyrogenes	Japan	23
41	UI 08452	interrogans	Unknown	Unknown	Laos	26
42	UI 08561	interrogans	Bataviae	Bataviae	Laos	27
43	UI 09600	interrogans	Unknown	Unknown	Laos	28
44	UI 12621	interrogans	Unknown	Unknown	Laos	29
45	UI 12758	interrogans	Unknown	Unknown	Laos	30
46	UI 12764	interrogans	Grippotyphosa	Grippotyphosa	Laos	31
100	2008720117	interrogans	Jalna	Australis	Croatia	69
105	Verdun HP	interrogans	Icterohaemorrhagiae	Icterohaemorrhagiae	France	None
123	Brem 137	interrogans	Bratislava	Australis	Germany	69
132	P2422	interrogans	Icterohaemorrhagiae	Icterohaemorrhagiae	Netherlands	6
133	P2518	interrogans	Copenhageni	Icterohaemorrhagiae	Netherlands	6
137	P2431	interrogans	Copenhageni	Icterohaemorrhagiae	Portugal	6
138	P2655	interrogans	Canicola	Canicola	Portugal	28
139	Sri Lanka 14	interrogans	Pyrogenes	Pyrogenes	Sri Lanka	74
143	Hond-utrecht	interrogans	Canicola	Canicola	Netherlands	28
144	Wijnberg	interrogans	Copenhageni	Icterohaemorrhagiae	Brazil	6
145	SR61	interrogans	Lai	Icterohaemorrhagiae	Sri Lanka	75
146	SriLanka1	interrogans	Pyrogenes	Pyrogenes	Sri Lanka	76
147	SriLanka2	interrogans	Pyrogenes	Pyrogenes	Sri Lanka	77
159	201600657	interrogans	Unknown	Unknown	Mayotte	81
166	200701872	interrogans	Pyrogenes	Pyrogenes	Mayotte	None
167	200901489	interrogans	Unknown	Pyrogenes	Mayotte	81
206	Fox 32256	interrogans	Pomona	Pomona	USA	5
207	2001025091	interrogans	Copenhageni	Icterohaemorrhagiae	USA	6

208	2002009669	interrogans	Copenhageni	Icterohaemorrhagiae	USA	6
210	CSL4002	interrogans	Unknown	Pomona	USA	5
211	PigK151	interrogans	Bratislava	Australis	USA	69
212	CSL10083	interrogans	Unknown	Pomona	USA	5
228	LT2156	interrogans	Zanoni	Pyrogenes	Australia	106
230	Szwajizak	interrogans	Szwajizak	Mini	Australia	107
231	Valbuzzi	interrogans	Valbuzzi	Grippotyphosa	Australia	108
235	acegua	interrogans	Muenchen	Australis	Brazil	69
236	Norma	interrogans	Hardjo	Sejroe	Brazil	19
237	Kantorowic	interrogans	Icterohaemorrhagiae	Icterohaemorrhagiae	Brazil	6
238	Kito	interrogans	Unknown	Canicola	Brazil	28
239	RCA	interrogans	Unknown	Unknown	Brazil	6
240	Prea	interrogans	Unknown	Unknown	Brazil	6
243	Fiocruz LV4117	interrogans	Unknown	Unknown	Brazil	6
244	Naam	interrogans	Naam	Icterohaemorrhagiae	Brazil	112
245	Wijnberg	interrogans	Copenhageni	Icterohaemorrhagiae	Brazil	6
248	LO-3	interrogans	Canicola	Canicola	Brazil	28
250	Fiocruz R83	interrogans	Copenhageni	Icterohaemorrhagiae	Brazil	6
264	R103	interrogans	Copenhageni	Icterohaemorrhagiae	Colombia	6
267	2006007831	interrogans	Copenhageni	Icterohaemorrhagiae	Guyana	6
268	HAI0024	interrogans	Unknown	Unknown	Peru	4
273	MMD1562	interrogans	Copenhageni	Icterohaemorrhagiae	Peru	6
288	HAI1594	interrogans	Unknown	Unknown	Peru	6
295	201502613	interrogans	Unknown	Canicola	Venezuela	28
296	201502616	interrogans	Unknown	Bataviae	Venezuela	4
299	Andaman	interrogans	Grippotyphosa	Grippotyphosa	Andaman Islands	2
301	201203348	interrogans	Canicola	Canicola	France	28
302	2002000634	interrogans	Copenhageni	Icterohaemorrhagiae	USA	6
307	Duyster	interrogans	Valbuzzi	Grippotyphosa	Unknown	108
327	201600677	interrogans	Unknown	Pyrogenes	Mayotte	81
335	201601174	interrogans	Icterohaemorrhagiae	Icterohaemorrhagiae	France	6
340	201601167	interrogans	Unknown	Pomona	France	5
341	201601168	interrogans	Unknown	Pomona	France	5
343	201601170	interrogans	Unknown	Pomona	France	5
344	201601171	interrogans	Unknown	Pomona	France	5
345	201601172	interrogans	Unknown	Pomona	France	5
347	201601175	interrogans	Icterohaemorrhagiae	Icterohaemorrhagiae	France	6
354	201601332	interrogans	Unknown	Unknown	Venezuela	37
372	IP1509008	interrogans	Unknown	Unknown	Uruguay	5
373	IP1509009	interrogans	Unknown	Pomona	Uruguay	5
374	IP1512011	interrogans	Unknown	Pomona	Uruguay	5

381	201700039	interrogans	Unknown	Pomona	USA	5
382	201600667	interrogans	Unknown	Pyrogenes	Mayotte	81
387	wtmanilae	interrogans	Manilae	Pyrogenes	Philippines	23
390	201703071	interrogans	Unknown	Unknown	Malaysia	4
393	201601010	interrogans	Unknown	Pyrogenes	Mayotte	81
400	201601332	interrogans	Unknown	Unknown	Venezuela	37
401	201601335	interrogans	Unknown	Unknown	New Caledonia	166
404	201700047	interrogans	Hardjobovis	Sejroe	England	19
405	201700048	interrogans	Hardjobovis	Sejroe	England	19
406	201700049	interrogans	Hardjoprajitno	Sejroe	Indonesia	19
408	200205138	interrogans	Unknown	Pomona	Croatia	5
411	201700041	interrogans	Kennewicki	Pomona	USA	5
417	201601617	interrogans	Unknown	Pomona	Unknown	5
418	201700043	interrogans	Unknown	Pomona	New Caledonia	5
421	hawain1295	interrogans	Hawain	Australis	New Guinea	174
422	fugis1186	interrogans	Fugis	Australis	Malaysia	175
423	jalna1187	interrogans	Jalna	Australis	Czechoslovakia	69
424	fugis1227	interrogans	Fugis	Australis	Malaysia	175
425	bangkok1233	interrogans	Bangkok	Australis	Thailand	176
426	hawain1238	interrogans	Hawain	Australis	New Guinea	174
430	lora1315	interrogans	Lora	Australis	Italy	180
431	muenchen1327	interrogans	Muenchen	Australis	Germany	69
452	interroganswewak1432	interrogans	Wewak	Australis	New Guinea	189
456	201700306	interrogans	Unknown	Pomona	Italy	5
458	201700316	interrogans	Unknown	Pomona	Italy	5
462	201700303	interrogans	Unknown	Pomona	Italy	5
463	201700305	interrogans	Unknown	Pomona	Italy	5
468	IP1509010	interrogans	Unknown	Pomona	Uruguay	5
469	IP1603018	interrogans	Unknown	Unknown	Uruguay	5
471	IP1609022	interrogans	Unknown	Pomona	Uruguay	5
472	IP1610023	interrogans	Unknown	Pomona	Uruguay	5
475	IP1611026	interrogans	Unknown	Pomona	Uruguay	5
477	IP1703028	interrogans	Unknown	Unknown	Uruguay	5
1000	200804418	interrogans	icterohaemorrhagiae	Icterohaemorrhagiae	France	6
1001	201103540	interrogans	icterohaemorrhagiae	icterohaemorrhagiae	France	6
1002	201300035	interrogans	icterohaemorrhagiae	icterohaemorrhagiae	France	6
1003	200210329	interrogans	icterohaemorrhagiae	icterohaemorrhagiae	France	6
1004	200412424	interrogans	icterohaemorrhagiae	icterohaemorrhagiae	France	6
1005	200704251	interrogans	icterohaemorrhagiae	icterohaemorrhagiae	France	6
1008	201902222	interrogans	Unknown	Australis	France	69
1009	201902223	interrogans	Unknown	Australis	France	69
4	2006006956	interrogans	Pyrogenes	Pyrogenes	Egypt	3

16	Sri Lanka 39	interrogans	Pyrogenes	Pyrogenes	Sri Lanka	10
168	201501067	interrogans	Unknown	Pyrogenes	Mayotte	81
269	HAI0156	interrogans	Unknown	Unknown	Peru	28
441	grippotyphosa1231	interrogans	Grippotyphosa	Grippotyphosa	Andaman Islands	2
443	valbuzzi1427	interrogans	Valbuzzi	Grippotyphosa	Australia	108
446	muelleri1448	interrogans	Muelleri	Grippotyphosa	Malaysia	187
447	grippotyphosa1481	interrogans	Grippotyphosa	Grippotyphosa	Andaman Islands	2
455	201700301	interrogans	Unknown	Pomona	Italy	5

[1] identification number (id) of the reference genome in BIGSdb (www.bigsdb.pasteur.fr)
[2] Clonal Group (CG) as defined by our cgMLST scheme (Guglielmini et al. 2019)

Supplementary Table 2 : Database of *Leptospira* core genomes used for variant calling

id[1]	isolate	species	serovar	serogroup	country	CG[2]
3	2006006986	interrogans	Grippotyphosa	Grippotyphosa	Egypt	2
299	Andaman	interrogans	Grippotyphosa	Grippotyphosa	Andaman Islands	2
11	2006006973	interrogans	Pyrogenes	Pyrogenes	Egypt	3
1052	Ag14_2020	interrogans	Pyrogenes	Pyrogenes	France	3
33	Swart	interrogans	Bataviae	Bataviae	Indonesia	4
1035	Ag03_2020	interrogans	Bataviae	Bataviae	France	4
206	Fox 32256	interrogans	Pomona	Pomona	USA	5
932	201900337	interrogans	Pomona	Pomona	France	5
97	RGA	interrogans	Icterohaemorrhagiae	Icterohaemorrhagiae	Belgium	6
106	Verdun LP	interrogans	Icterohaemorrhagiae	Icterohaemorrhagiae	France	6
144	Wijnberg	interrogans	Copenhageni	Icterohaemorrhagiae	Brazil	6
246	Fiocruz L1-130	interrogans	Copenhageni	Icterohaemorrhagiae	Brazil	6
13	pyrogenes	borgpetersenii	Unknown	Pyrogenes	Nigeria	7
14	TE 0159	borgpetersenii	Kenya	Ballum	Tanzania	8
19	Sh9	borgpetersenii	Kenya	Ballum	Tanzania	8
15	Sri Lanka 30	interrogans	Pyrogenes	Pyrogenes	Sri Lanka	9
16	Sri Lanka 39	interrogans	Pyrogenes	Pyrogenes	Sri Lanka	10
17	Sri Lanka 46	interrogans	Pyrogenes	Pyrogenes	Sri Lanka	11
18	TE 1992	interrogans	Lora	Australis	Tanzania	12
86	200801910	borgpetersenii	Castellonis	Ballum	Guadeloupe	15
233	Noumea 25	borgpetersenii	Unknown	Ballum	New Caledonia	15
27	56601	interrogans	Lai	Icterohaemorrhagiae	China	16
25	56609	interrogans	Linhai	Grippotyphosa	China	17
29	P2653	weilii	Unknown	Unknown	China	18
31	Hardjoprajitno	interrogans	Hardjo	Sejroe	Indonesia	19

236	Norma	interrogans	Hardjo	Sejroe	Brazil	19
34	3522 CT	kirschneri	Cynopteri	Cynopteri	Indonesia	20
36	JB	kirschneri	Unknown	Unknown	Iran	22
403	201700045	kirschneri	Unknown	Grippotyphosa	Turkey	22
37	UP-MMC-NIID HP	interrogans	Manilae	Pyrogenes	Japan	23
39	UI 09149	borgpetersenii	Unknown	Unknown	Laos	24
73	UT234	interrogans	Bataviae	Bataviae	Thailand	26
42	UI 08561	interrogans	Bataviae	Bataviae	Laos	27
143	Hond-utrecht	interrogans	Canicola	Canicola	Netherlands	28
939	H-645.10	interrogans	Canicola	Canicola	Cuba	28
44	UI 12621	interrogans	Unknown	Unknown	Laos	29
70	P2529	interrogans	Bim	Autumnalis	Thailand	30
768	12758	interrogans	Unknown	Autumnalis	Laos	30
46	UI 12764	interrogans	Grippotyphosa	Grippotyphosa	Laos	31
49	LNT 1234	weilii	Unknown	Unknown	Laos	32
50	UI 13098	weilii	Unknown	Unknown	Laos	33
51	Langkawi	interrogans	Lai	Icterohaemorrhagiae	Malaysia	34
52	Bejo-Iso9	kmetyi	Unknown	Unknown	Malaysia	35
974	201900343	interrogans	Djasiman	Djasiman	Unknown	37
60	FPW1039	interrogans	Unknown	Unknown	Thailand	38
62	L0374	interrogans	Pyrogenes	Pyrogenes	Thailand	39
66	L1111	interrogans	Bataviae	Bataviae	Thailand	40
783	14721	interrogans	Unknown	Bataviae	Laos	40
72	UT126	interrogans	Unknown	Unknown	Thailand	41
77	H2	kirschneri	Unknown	Unknown	Thailand	42
78	UT130	kirschneri	Grippotyphosa	Grippotyphosa	Thailand	43
79	2006001853	weilii	Unknown	Unknown	Thailand	44
80	2006001855	weilii	Unknown	Unknown	Thailand	45
81	LNT 1194	weilii	Unknown	Unknown	Thailand	46
82	UI 14631	weilii	Unknown	Unknown	Thailand	47
737	14631	weilii	Unknown	Unknown	Laos	47
84	1051	kirschneri	Bim	Autumnalis	Barbados	49
213	PUO 1247	kirschneri	Bim	Autumnalis	USA	49
88	200702252	santarosai	Unknown	Unknown	Guadeloupe	50
89	200403458	santarosai	Unknown	Unknown	Guadeloupe	50
90	ST188	santarosai	Unknown	Unknown	Trinidad and Tobago	51
91	7	santarosai	Arenal	Javanica	Costa Rica	52
92	11	santarosai	Arenal	Javanica	Costa Rica	53
93	MAVJ 401	santarosai	Arenal	Javanica	Costa Rica	54
94	CZ 214T	noguchii	Panama	Panama	Panama	55
1040	Ag11_2020	noguchii	Panama	Panama	France	55

96	LT821	santarosai	Shermani	Shermani	Panama	56
1066	Ag22_2020	santarosai	Unknown	Unknown	France	56
98	Nikolaevo	kirschneri	Bulgarica	Autumnalis	Bulgaria	57
101	2008720114	kirschneri	Unknown	Unknown	Croatia	59
110	200702274	kirschneri	Valbuzzi	Grippotyphosa	France	62
315	dessain	kirschneri	Grippotyphosa	Grippotyphosa	France	62
112	200801925	kirschneri	Unknown	Mini	Mayotte	63
113	200802841	kirschneri	Unknown	Unknown	Mayotte	64
117	201402975	kirschneri	Grippotyphosa	Grippotyphosa	France	65
684	17/OD2023	borgpetersenii	Unknown	Sejroe	Switzerland	68
1056	Ag15_2020	borgpetersenii	unknown	sejroe	France	68
99	2008720116	interrogans	Jalna	Australis	Croatia	69
123	Brem 137	interrogans	Bratislava	Australis	Germany	69
124	Brem 179	kirschneri	Valbuzzi	Grippotyphosa	Germany	70
214	RM52	kirschneri	Grippotyphosa	Grippotyphosa	USA	70
129	Sponselee CDC	borgpetersenii	Hardjo	Sejroe	Netherlands	72
225	L550	borgpetersenii	Hardjobovis	Sejroe	Australia	72
994	203	borgpetersenii	Hardjo-bovis	not examined	USA	72
1054	Ag08_2020	borgpetersenii	unknown	sejroe	France	72
140	Vehlefans 3	kirschneri	Mozdok	Pomona	Portugal	73
251	61H	kirschneri	Mozdok	Pomona	Brazil	73
686	FMAS_AW1	interrogans	Unknown	Autumnalis	Sri Lanka	74
145	SR61	interrogans	Lai	Icterohaemorrhagiae	Sri Lanka	75
146	SriLanka1	interrogans	Pyrogenes	Pyrogenes	Sri Lanka	76
147	SriLanka2	interrogans	Pyrogenes	Pyrogenes	Sri Lanka	77
163	201000851	borgpetersenii	Mini	Mini	Mayotte	78
149	200901116	mayottensis	Unknown	Mini	Mayotte	79
162	200901868	borgpetersenii	Pomona	Pomona	Mayotte	80
382	201600667	interrogans	Unknown	Pyrogenes	Mayotte	81
338	201601027	mayottensis	Unknown	Unknown	Mayotte	82
175	200803703	kirschneri	Unknown	Mini	Mayotte	83
172	200901119	kirschneri	Unknown	Mini	Mayotte	84
174	200801774	kirschneri	Unknown	Grippotyphosa	Mayotte	85
205	JB197	borgpetersenii	Hardjobovis	Sejroe	USA	98
612	IP1711048	borgpetersenii	Unknown	Unknown	Uruguay	98
215	2006001870	noguchii	Unknown	Unknown	USA	99
217	2001034031	noguchii	Unknown	Unknown	USA	100
219	Oregon	santarosai	Szwajizak	Mini	USA	101
220	2000027870	santarosai	Unknown	Unknown	USA	102
221	2000030832	santarosai	Unknown	Unknown	USA	103
223	Ecochallenge	weilii	Unknown	Hebdomadis	USA	104
228	LT2156	interrogans	Zanoni	Pyrogenes	Australia	106

230	Szwajizak	interrogans	Szwajizak	Mini	Australia	107
231	Valbuzzi	interrogans	Valbuzzi	Grippotyphosa	Australia	108
307	Duyster	interrogans	Valbuzzi	Grippotyphosa	Unknown	108
232	LT2116	weilii	Topaz	Tarassovi	Australia	109
244	Naam	interrogans	Naam	Icterohaemorrhagiae	Brazil	112
253	U73	noguchii	Unknown	Panama	Brazil	113
380	201700036	noguchii	Unknown	Panama	Brazil	113
254	Hook	noguchii	Unknown	Australis	Brazil	114
255	Bonito	noguchii	Unknown	Autumnalis	Brazil	115
256	Cascata	noguchii	Unknown	Bataviae	Brazil	116
257	U160	santarosai	Unknown	Unknown	Brazil	117
258	U164	santarosai	Unknown	Unknown	Brazil	118
259	U233	santarosai	Unknown	Unknown	Brazil	119
261	Fiocruz LV3954	santarosai	Unknown	Unknown	Brazil	120
262	Fiocruz LV4135	santarosai	Unknown	Unknown	Brazil	120
266	JET	santarosai	Unknown	Unknown	Colombia	122
275	MMD1493	kirschneri	Unknown	Unknown	Peru	123
280	ZUN142	noguchii	Autumnalis	Autumnalis	Peru	125
281	CBC1416	santarosai	Unknown	Unknown	Peru	126
282	CBC523	santarosai	Unknown	Unknown	Peru	127
283	CBC1531	santarosai	Unknown	Unknown	Peru	128
284	CBC613	kirschneri	Unknown	Unknown	Peru	129
410	201700037	kirschneri	Galtoni	Canicola	Argentina	129
285	HAI134	santarosai	Unknown	Unknown	Peru	130
286	HAI1349	santarosai	Unknown	Unknown	Peru	131
289	HAI821	santarosai	Unknown	Unknown	Peru	132
290	MOR084	santarosai	Unknown	Unknown	Peru	133
291	CBC379	santarosai	Unknown	Unknown	Peru	134
304	B 81/7 type 3	kirschneri	Tsaratsovo	Pomona	Bulgaria	139
637	89/90	kirschneri	Unknown	Pomona	France	139
325	201600675	borgpetersenii	Unknown	Unknown	Mayotte	145
729	201701208	borgpetersenii	Unknown	Unknown	Mayotte	145
376	IP1512017	noguchii	Unknown	Unknown	Uruguay	162
377	201602279	weilii	Unknown	Unknown	France	163
386	201700040	borgpetersenii	Szwajizak	Mini	USA	165
401	201601335	interrogans	Unknown	Unknown	New Caledonia	166
402	201700042	borgpetersenii	Guidae	Tarassovi	Brazil	167
407	201700038	noguchii	Peruviana	Australis	Peru	168
421	1295	interrogans	Hawain	Australis	New Guinea	174
422	1186	interrogans	Fugis	Australis	Malaysia	175
425	1233	interrogans	Bangkok	Australis	Thailand	176
427	1252	noguchii	Soteropolitana	Australis	Brazil	177

428	1252	kirschneri	Ramisi	Australis	Kenya	178
498	1255	kirschneri	Ramisi	Australis	Kenya	178
429	bajan1256	noguchii	Bajan	Australis	Barbados	179
449	barbudensis1433	noguchii	Barbudensis	Australis	Barbados	179
430	lora1315	interrogans	Lora	Australis	Italy	180
945	201900325	interrogans	Australis	Australis	France	180
432	nicaragua1345	noguchii	Nicaragua	Australis	Nicaragua	181
440	vanderhoedeni1226	kirschneri	Vanderhoedeni	Grippotyphosa	Israel	184
499	1226	kirschneri	Vanderhoedeni	Grippotyphosa	Israel	184
1176	Wumalasena	kirschneri	Ratnapura	Grippotyphosa	India	185
445	canalzonae1440	santarosai	Canalzonae	Grippotyphosa	Panama	186
446	muelleri1448	interrogans	Muelleri	Grippotyphosa	Malaysia	187
515	1294	noguchii	Unknown	Australis	Panama	188
452	interroganswewak1432	interrogans	Wewak	Australis	New Guinea	189
509	1432	interrogans	Wewak	Australis	New Guinea	189
473	IP1611024	noguchii	Unknown	Unknown	Uruguay	192
474	IP1611025	noguchii	Unknown	Unknown	Uruguay	193
476	IP1703027	noguchii	Unknown	Unknown	Uruguay	194
835	201900342	santarosai	Unknown	Unknown	France	196
537	SSS1	kmetyi	Unknown	Unknown	Malaysia	201
541	SCS7	kmetyi	Unknown	Unknown	Malaysia	202
543	SCS11	kmetyi	Unknown	Unknown	Malaysia	202
544	SSS17	kmetyi	Unknown	Unknown	Malaysia	203
545	SSS18	kmetyi	Unknown	Unknown	Malaysia	203
559	201702412	kmetyi	Unknown	Unknown	Malaysia	203
595	201702448	kmetyi	Unknown	Unknown	Malaysia	215
599	IP1705032	noguchii	Unknown	Unknown	Uruguay	224
601	IP1708035	noguchii	Unknown	Unknown	Uruguay	225
603	IP1709037	noguchii	Unknown	Unknown	Uruguay	226
503	nogu rushan1311	noguchii	Rushan	Australis	China	227
929	201900348	noguchii	Louisiana	Louisiana	France	227
511	liangguang1445	interrogans	Unknown	Grippotyphosa	China	228
538	SSS2	kmetyi	Unknown	Unknown	Malaysia	233
539	SSS4	kmetyi	Unknown	Unknown	Malaysia	234
761	Strain 3705	interrogans	Wolffi	Sejroe	Indonesia	238
841	201900351	interrogans	not examined	Sejroe	France	238
618	Borisihin	interrogans	Unknown	Tarassovi	Russia	240
687	FMAS_RT1	weilii	Not examined	Unknown	Sri Lanka	262
689	201800566	interrogans	Unknown	Pyrogenes	Sri Lanka	263
692	201800569	interrogans	Unknown	Pyrogenes	Sri Lanka	263
690	FMAS_PD2	weilii	Not examined	Celledoni	Sri Lanka	264
691	FMAS_KG1	interrogans	Unknown	Bataviae	Sri Lanka	265

693	FMAS_AP1	interrogans	Unknown	Autumnalis	Sri Lanka	266
1026	FMAS_PN3	interrogans	not examined	Autumnalis	Sri Lanka	266
40	UI 09931	borgpetersenii	Javanica	Javanica	Laos	267
1029	202000078	borgpetersenii	not examined	Unknown	Sri Lanka	267
699	201800563	interrogans	Unknown	Autumnalis	Sri Lanka	269
702	201800565	interrogans	Unknown	Autumnalis	Sri Lanka	271
718	12539	interrogans	Unknown	Autumnalis	Laos	272
733	12268	interrogans	Unknown	Autumnalis	Laos	272
723	LNT 2340	weilii	Unknown	Unknown	Laos	273
736	14535	weilii	Unknown	Unknown	Laos	274
745	27845	interrogans	Unknown	Australis	Laos	275
746	8434	interrogans	Unknown	Grippotyphosa	Laos	276
747	12769	interrogans	Unknown	Grippotyphosa	Laos	277
800	8368	interrogans	Unknown	Grippotyphosa	Laos	277
753	23152	interrogans	Unknown	Bataviae	Laos	278
754	LNT 1600	interrogans	Unknown	Bataviae	Laos	279
755	LNT 2859	interrogans	Unknown	Icterohaemorrhagiae	Laos	280
756	LNT 3086	interrogans	Unknown	Grippotyphosa	Laos	281
757	LNT 3110	weilii	Unknown	Unknown	Laos	282
758	SV 725	weilii	Unknown	Unknown	Laos	283
759	SV 865	interrogans	Unknown	Pomona	Laos	284
788	EFS SV 588	interrogans	Unknown	Pomona	Laos	284
763	UI29907	interrogans	Unknown	Bataviae	Laos	285
764	UI36788	interrogans	Unknown	Grippotyphosa	Laos	286
771	UI15191	interrogans	Unknown	Pomona	Laos	287
809	UI19893	interrogans	Unknown	Unknown	Laos	288
774	SV719	interrogans	Unknown	Icterohaemorrhagiae	Laos	289
780	UI37443	interrogans	Unknown	Icterohaemorrhagiae	Laos	290
781	201800560	interrogans	Unknown	Autumnalis	Sri Lanka	291
986	FMAS_KW2	interrogans	not examined	not examined	Sri Lanka	291
785	LNT 2987	weilii	Unknown	Unknown	Laos	292
787	SV 1038	weilii	Unknown	Unknown	Laos	293
1069	202000717	interrogans	Not examined	Grippotyphosa	France	294
802	14346	interrogans	Unknown	Grippotyphosa	Laos	295
806	LNT1612	interrogans	Unknown	Bataviae	Laos	296
807	LNT2714	interrogans	Unknown	Autumnalis	Laos	297
808	LNT 3077	interrogans	Unknown	Grippotyphosa	Laos	298
810	UI33938	kirschneri	Unknown	Unknown	Laos	299
811	UI37640	interrogans	Unknown	Unknown	Laos	300
825	8440	interrogans	Unknown	Autumnalis	Laos	302
829	EFS SV 547	interrogans	Unknown	Pyrogenes	Laos	303
834	201900326	interrogans	Unknown	Unknown	France	304

1034	Ag02_2020	interrogans	Autumnalis	Autumnalis	France	304
700	201900331	kirschneri	None	Grippotyphosa	France	305
842	201900349	interrogans	None	Bratislava	France	306
1049	ACSBRA_2020	interrogans	Bratislava	Australis	France	306
849	201801174	interrogans	Unknown	Unknown	Laos	307
927	201900345	weilii	Sarmin	Sarmin	France	308
1155	20210884	weilii	sarmin	Sarmin	France	308
928	201900347	borgpetersenii	Javanica	Javanica	France	309
1047	Ag23_2020	borgpetersenii	Javanica	Javanica	France	309
944	201902908	santarosai	Unknown	Tarassovi	Martinique	310
950	201900466	interrogans	Unknown	Sejroe	France	311
894	MM0270CK	borgpetersenii	Balcanica	Sejroe	New Zealand	312
926	RL0016	borgpetersenii	Balcanica	Sejroe	New Zealand	312
956	Leptospira-5	borgpetersenii	not examined	Javanica	Russia	313
957	Leptospira-34	borgpetersenii	not examined	Javanica	Russia	313
975	201900350	borgpetersenii	Sejroe	Sejroe	Unknown	318
1059	ACSSAX2020	borgpetersenii	unknown	saxkoebing	France	318
1018	201902972	weilii	Unknown	Hebdomadis	France	320
1019	201902973	weilii	Unknown	Hebdomadis	France	320
987	FMAS_KW1	interrogans	not examined	not examined	Sri Lanka	321
1022	C17	interrogans	not examined	Pyrogenes	Sri Lanka	321
1025	C20	interrogans	not examined	Icterohaemorrhagiae	Sri Lanka	322
1030	C25	kirschneri	not examined	Grippotyphosa	Sri Lanka	323
1055	Ag09_2020	interrogans	unknown	hebdomadis	France	331
1070	202001123	santarosai	Not examined	Sejroe	France	338
1071	202001124	santarosai	Not examined	Sejroe	France	338
1085	202001526	interrogans	Not examined	Australis	France	345
1148	202100566	interrogans	Not examined	Australis	France	345
1086	200702592	weilii	Not examined	Not examined	Thailand	346
1099	GAF646	borgpetersenii	Unknown	Unknown	France	348
1101	LNA10	interrogans	canicola	Canicola	Madagascar	349
1114	GAF1022	borgpetersenii	Not examined	Not examined	France	352
1116	RM 1	kirschneri	Sokoine	Icterohaemorrhagiae	Unknown	353
1118	Mwogolo	kirschneri	Unknown	Icterohaemorrhagiae	Unknown	354
1119	Lambwe	kirschneri	Unknown	Autumnalis	Unknown	355
1120	Kwale	borgpetersenii	Unknown	Pyrogenes	Unknown	356
988	LS 001/16	kmetyi	not examined	not examined	Malaysia	365
993	VS2413	mayottensis	not examined	not examined	Madagascar	366
1183	IP1712055	noguchii	Not examined	Not examined	Uruguay	385

[1] identification number (id) of the reference genome in BIGSdb (www.bigsdb.pasteur.fr)
[2] Clonal Group (CG) as defined by our cgMLST scheme (Guglielmini et al. 2019)

Supplementary Table 3 : Genes showing SNPs and INDELs. The genome of L. interrogans serovar Copenhageni strain Fiocruz L1-130 (id246) was compared with the sequences from samples which were assigned to L. interrogans serovars Copenhageni and Icterohaemorrhagiae (Tables 1 and 2).

Supplementary Table 3 : Genes showing SNPs and INDELs. The genome of L. interrogans serovar Copenhageni strain Fiocruz L1-130 (id246) was compared with the sequences from samples which were assigned to L. interrogans serovars Copenhageni and Icterohaemorrhagiae (Tables 1 and 2).

id	gene	location fasta	ref	alter	effect	mutation type	impact	CDS position	codon change	protein effect	protein size	annotation	Putative function	COG
246	LIC10121	373	G	A	missense_variant	MISSENSE	MODERATE	373G>A	Ggg/Agg	Gly125Arg	318	rsgA	Putative ribosome biogenesis GTPase RsgA	Translation, ribosomal structure and biogenesis
246	LIC10339	2208	G	A	synonymous_variant	SILENT	LOW	2208G>A	gtG/gtA	Val736Val	846	clpC	ATP-dependent Clp protease ATP-binding subunit	Posttranslational modification, protein turnover, chaperones
246	LIC10484	1124	C	T	missense_variant	MISSENSE	MODERATE	1124C>T	gCt/gTt	Ala375Val	449	thrC	threonine synthase	Amino acid transport and metabolism
246	LIC10580	630	G	A	synonymous_variant	SILENT	LOW	630G>A	ttG/ttA	Leu210Leu	631	msbA	ATP-dependent lipid A-core flippase	Defense mechanisms
246	LIC10842	903	G	A	synonymous_variant	SILENT	LOW	903G>A	ctG/ctA	Leu301Leu	307	dapA	Dihydrodipicolinate synthase	Amino acid transport and metabolism
246	LIC10874	294	C	G	missense_variant	MISSENSE	MODERATE	294C>G	caC/caG	His98Gln	1035		molybdopterin oxidoreductase	Energy production and conversion
246	LIC12841	221	G	C	missense_variant	MISSENSE	MODERATE	221G>C	gGa/gCa	Gly74Ala	434	dapL	LL-diaminopimelate aminotransferase	Amino acid transport and metabolism
246	LIC11070	931	G	A	missense_variant	MISSENSE	MODERATE	931G>A	Gcc/Acc	Ala311Thr	388	dhaT	1,3-propanediol dehydrogenase	Energy production and conversion
246	LIC11265	108	T	C	synonymous_variant	SILENT	LOW	108T>C	ggT/ggC	Gly36Gly	75		hypothetical protein	Function unknown
246	LIC11311	624	A	G	synonymous_variant	SILENT	LOW	624A>G	gtA/gtG	Val208Val	385		acetyl-CoA C-acyltransferase	Lipid transport and metabolism
246	LIC11335	1587	C	T	synonymous_variant	SILENT	LOW	1587C>T	aaC/aaT	Asn529Asn	546	groL	60 kDa chaperonin	Posttranslational modification, protein turnover, chaperones
246	LIC11374	13	A	G	missense_variant	MISSENSE	MODERATE	13A>G	Atc/Gtc	Ile5Val	259	fliR	flagellar biosynthetic protein	Cell motility
246	LIC12764	667	G	A	missense_variant	MISSENSE	MODERATE	667G>A	Ggc/Agc	Gly223Ser	569	ilvB	acetolactate synthase I large subunit	Amino acid transport and metabolism
246	LIC11455	1985	G	T	missense_variant	MISSENSE	MODERATE	1985G>T	aGg/aTg	Arg662Met	1186	mfd	transcription-repair coupling factor	Transcription
246	LIC11518	1929	C	T	synonymous_variant	SILENT	LOW	1929C>T	caC/caT	His643His	914		biotin carboxylase subunit of acetyl CoA carboxylase	Lipid transport and metabolism
246	LIC11570	1820	A	G	missense_variant	MISSENSE	MODERATE	1820A>G	gAa/gGa	Glu607Gly	615	pulD	Secretin PulD	Intracellular trafficking, secretion, and vesicular transport
246	LIC11604	676	GAAAAAGAAA	GAAAAAAGAAA	frameshift_variant	INSERTION	HIGH	681dupA	gaa/Agaa	Glu228fs	276		hypothetical protein	Function unknown
246	LIC11791	1374	C	T	synonymous_variant	SILENT	LOW	1374C>T	gaC/gaT	Asp458Asp	569	recG	ATP-dependent DNA helicase	Transcription
246	LIC12955	31	A	G	missense_variant	MISSENSE	MODERATE	31A>G	Aaa/Gaa	Lys11Glu	153	fruA	PTS system fructose-specific EIIA component	Carbohydrate transport and metabolism
246	LIC10974	550	G	T	missense_variant	MISSENSE	MODERATE	550G>T	Gcc/Tcc	Ala184Ser	421	fadA	3-ketoacyl-CoA thiolase	Lipid transport and metabolism
246	LIC13481	722	A	G	missense_variant	MISSENSE	MODERATE	722A>G	aAa/aGa	Lys241Arg	269	ycfH	Uncharacterized metal-dependent hydrolase	Replication, recombination and repair
246	LIC12870	705	G	T	synonymous_variant	SILENT	LOW	705G>T	ggG/ggT	Gly235Gly	279	rplB	50S ribosomal protein L2	Translation, ribosomal structure and biogenesis
